# Detecting Prognosis Risk Biomarkers for Colon Cancer Through Multi-Omics-Based Prognostic Analysis and Target Regulation Simulation Modeling

**DOI:** 10.3389/fgene.2020.00524

**Published:** 2020-05-26

**Authors:** Zuojing Yin, Xinmiao Yan, Qiming Wang, Zeliang Deng, Kailin Tang, Zhiwei Cao, Tianyi Qiu

**Affiliations:** ^1^Department of Gastroenterology, Shanghai Tenth People’s Hospital, College of Life Science and Technology, Tongji University, Shanghai, China; ^2^Shanghai Public Health Clinical Center, Fudan University, Shanghai, China

**Keywords:** colon cancer, prognostic analysis, multi-omics analysis, *in silico* simulation, pathway integration

## Abstract

**Background:**

Colon cancer is one of the most common health threats for humans since its high morbidity and mortality. Detecting potential prognosis risk biomarkers (PRBs) is essential for the improvement of therapeutic strategies and drug development. Currently, although an integrated prognostic analysis of multi-omics for colon cancer is insufficient, it has been reported to be valuable for improving PRBs’ detection in other cancer types.

**Aim:**

This study aims to detect potential PRBs for colon adenocarcinoma (COAD) samples through the cancer genome atlas (TCGA) by integrating muti-omics.

**Materials and Methods:**

The multi-omics-based prognostic analysis (MPA) model was first constructed to systemically analyze the prognosis of colon cancer based on four-omics data of gene expression, exon expression, DNA methylation and somatic mutations on COAD samples. Then, the essential features related to prognosis were functionally annotated through protein–protein interaction (PPI) network and cancer-related pathways. Moreover, the significance of those essential prognostic features were further confirmed by the target regulation simulation (TRS) model. Finally, an independent testing dataset, as well as the single cell-based expression dataset were utilized to validate the generality and repeatability of PRBs detected in this study.

**Results:**

By integrating the result of MPA modeling, as well the PPI network, integrated pathway and TRS modeling, essential features with gene symbols such as EPB41, PSMA1, FGFR3, MRAS, LEP, C7orf46, LOC285000, LBP, ZNF35, SLC30A3, LECT2, RNF7, and DYNC1I1 were identified as PRBs which provide high potential as drug targets for COAD treatment. Validation on the independent testing dataset demonstrated that these PRBs could be applied to distinguish the prognosis of COAD patients. Moreover, the prognosis of patients with different clinical conditions could also be distinguished by the above PRBs.

**Conclusions:**

The MPA and TRS models constructed in this paper, as well as the PPI network and integrated pathway analysis, could not only help detect PRBs as potential therapeutic targets for COAD patients but also make it a paradigm for the prognostic analysis of other cancers.

## Introduction

As one of the most common cancer types and the second leading cause of cancer mortality (Hernandez et al., 2014), colorectal cancer (CRC) is highly prevalent worldwide, with more than 1.2 million new cases and over 600 thousand deaths each year (Li et al., 2015). Even though nearly 60% of CRC patients can be treated through therapeutic surgical resection and adjuvant chemotherapy, approximately 20–30% of patients will eventually suffer from disease recurrence and experience poor prognosis (O’Connell et al., 2008; Andre et al., 2009). The diagnosis and prognosis of CRC, especially its branch colon cancer (Marley and Nan, 2016), has received much attention in recent researches. Thus, approaches which could efficiently identify the PRBs for colon cancer with diagnosis, monitoring, and prognosis are highly desired to improve the cure rate and overall survival (OS) (Melichar, 2013; Zhou et al., 2018a, b).

With the development of next-generation sequencing (NGS), essential PRBS for colon cancer from sequencing data such as gene expression (Calon et al., 2015; Okugawa et al., 2017), exon expression (Katoh et al., 2015), DNA methylation status (Kandimalla et al., 2017), mutational profile (Yu et al., 2015; Taieb et al., 2016) and others (Zheng et al., 2001; Ozawa et al., 2017) were determined. For example, it was reported that CDX2 could be used as PRBs for stage II and stage III colon cancer (van den Braak et al., 2018). And, mutations on BRAF (V600E) and KRAS were significantly associated with disease-free survival (DFS) and OS in CRC patients with microsatellite-stable tumors (Taieb et al., 2016). Additionally, it was reported that high expression of hsa-mir-155 and low expression of hsa-let-7a-2 were correlated with poor survival in lung cancer (Yanaihara et al., 2006). Moreover, protein biomarkers such as CA19-9, CA 72-4 and carcinoembryonic antigen (CEA), can be used as PRBs of colorectal carcinoma (Zheng et al., 2001), and plasma vascular endothelial growth factor-A (VEGF-A) can be used as a PRBs for colon cancer (Luo and Xu, 2014). Despite all the above efforts, no non-invasive, specific, sensitive, and economical methods are reported to identify the PRBs for all types of CRC patients in clinical (Das et al., 2017). Existing PRBs are only sensitive for limited patients and fail to be extended for large-scale populations (Xie et al., 2018). Considering that the omics information from different patients are not consistent, it is necessary to apply multi-omics information in large-scale populations to detect general PRBs. PRBs from multi-omics rather than single one cannot only help the diagnosis of colon cancer but also increase sensitivity to conventional therapies and improve prognosis.

By taking advantage of The Cancer Genome Atlas (TCGA) program (Tomczak et al., 2015), multi-omics molecular profiles including transcriptome, exon expression, DNA methylation, mutations, etc. are collated along with clinical annotations for patients. In that case, it is possible to discover the PRBs with multi-omics information across large-scale populations by machine learning techniques (Cruz and Wishart, 2007; Kourou et al., 2015). In this study, prognostic analysis of COAD patients was performed by integrating multi-omics data which were closely associated with the expression or regulation of genes including gene expression, exon expression, DNA methylation and mutations derived from UCSC Xena database (Liu et al., 2017; Qu et al., 2017; Zhang X. et al., 2017), as well as clinical survival information of patients. Firstly, a MPA model was generated to identify essential features that significantly affect the prognosis of COAD patients. Then, the function of the above features was analyzed through the PPI network and pathway integration analysis. Moreover, the TRS model was provided to validate the significance of those essential features that alteration could increase the OS of COAD patients. By integrating the result of MPA modeling, as well as the PPI network, integrated pathway and TRS modeling, essential features with gene symbols of EPB41, PSMA1, FGFR3, MRAS, LEP, C7orf46, LOC285000, LBP, ZNF35, SLC30A3, LECT2, RNF7, and DYNC1I1 were detected as PRBs for COAD. The validation of the independent dataset showed that these detected PRBs could not only distinguish the prognosis of colon cancer patients from other data sources, but also reflect significant difference between tumor and normal cells from single-cell based expression profile. Moreover, these PRBs were also effectively distinguish the prognosis of patients with different clinical conditions. With the accumulation of multi-omics data and clinical information, it is possible for us to comprehensively investigate PRBs and perform therapeutic targets for future drug development.

## Materials and Methods

### Data Source

The overall survival (OS) of 551 COAD samples as the survival information is derived from the TCGA module of Public Xena Hubs in the UCSC Xena database (Liu et al., 2017; Qu et al., 2017; Zhang X. et al., 2017). Besides, the clinical information of COAD samples including age, weight, person neoplasm cancer status, number of first degree relatives with cancer diagnosis, etc. were downloaded from the phenotype section of the TCGA module in the UCSC Xena database. Four omics data were downloaded followed the same process, including gene expression profiles of 329 COAD samples, exon expression profiles of 329 COAD samples, DNA methylation profiles of 337 COAD samples and somatic mutation information of 217 COAD samples (Supplementary Table S1). For each patient, a tumor sample was selected as the research object by filtering out the samples from normal tissue according to the nomenclature of TCGA sample IDs.

All the profile dataset of four omics were downloaded from the UCSC Xena database. Briefly, level 3 gene expression profiles with 20,530 gene features recorded in the UCSC Xena database were experimentally generated using the Illumina HiSeq 2000 RNA sequencing platform (Prego-Faraldo et al., 2018) from the University of North Carolina TCGA genome characterization center. The stored exon expression profiles were generated using the same platform as the gene expression profiles, with 239,322 exon features. The downloaded DNA methylation profiles were obtained from the platform of Illumina Infinium HumanMethylation450 (Hong et al., 2019), which consists of 375,066 methylation features. Moreover, for somatic mutations, the recorded sequencing data were generated on the Illumina GA system containing 239,322 mutations. After obtained the original mutation profiles, the information for somatic mutations was integrated into a binary matrix, in which mutations at the corresponding position were marked as 1 or 0. Besides, the gene annotation information of exon and methylation were also derived from the TCGA module of Public Xena Hubs in the UCSC Xena database.

The background Protein–protein interaction (PPI) network used in this project contained 10,462 nodes and 55,317 interactions was constructed mainly based on three database, including HPRD version 9 (Stelzl et al., 2005), Mint version 2012 (Zanzoni et al., 2002) and IntAct version 4.2.12 (Hermjakob et al., 2004). And, biological pathways for enrichment and analysis were integrated from KEGG version 87.0 (Kanehisa et al., 2017) and GeneCards version 4.12 (Safran et al., 2010). Targets of drugs were retrieved from DrugBank version 5.0 (Wishart et al., 2018) and the TTD version 2018 (Li et al., 2018).

The independent dataset was obtained from the NCBI GEO database with the accession number of GSE17538, in which the expression profile including 54,675 probes in 177 colon cancer patients with survival information from Moffitt Cancer Center (Smith et al., 2010). The single-cell based RNA-seq dataset of colon cancer was downloaded from the NCBI GEO database with the accession number of GSE81861 (Li et al., 2017). In this dataset, single-cell sequencing data containing 11 primary colorectal tumors and matched normal mucosa (NM) cell with 57,240 genes are selected. Among them, four groups including all cell count (266 cells for NM and 375 cells for tumor), all cell FPKM (215 cells for NM and 375 cells in tumor), epithelial cell count (160 cells for NM and 272 cells in tumor), and epithelial cell FPKM (160 cells for NM and 272 cells for tumor) were collected.

### Determining Prognosis-Related Features or MPA Model Construction

For each omics dataset, the intersected tumor samples with the survival records were selected as the patient samples and further divided into high-OS group (positive) and low-OS group (negative) by setting the threshold of OS as 5 years (1,825 days) (Gustafsson et al., 2016). Further, two-tailed *T*-tests were used to evaluate the different features between positive and negative samples. For each omics profile, the top 1,000 features with *P*-values in ascending order were first screened and further filtered with conditions of *P* < 0.01 and fold change (FC) > 1.5 or FC < 2/3.

Then, to reduce the feature dimensionality of multi-omics profiles combined by single-omics, exploratory factor analysis (EFA) (Cole et al., 2018) was performed on the profile of the above detected differential features on single omics by using the psych package of R software (Lorenzo-Seva and Van Ginkel, 2016) to obtain the weight matrix between factors and original features, as well as the scoring matrix of factors. For multi-omics, including double-omics, triple-omics, and quadruple-omics, the factor scoring matrix was obtained by combining the corresponding factor scoring matrix in single-omics. Furthermore, the scoring matrix of factors in each omics dataset was integrated with logarithmically transformed OS, and unsupervised hierarchical clustering could be performed by using the pheatmap package of R software (Xu et al., 2018) to verify the classification performance of factors.

Then, to detect the essential features that might be closely associated with the prognosis of COAD in each single-omics profile, the weight matrix of factors obtained from the above EFA process was normalized from 0 to 1 and the weights of different features could be sorted in descending orders for each factor. Essential features with the maximum weight for each factor, which prompted the performance of distinguishing prognosis, were selected as the essential features in each single-omics for subsequent prognostic modeling. For multi-omics, the profiles of essential features were produced by integrating the corresponding ones in single-omics, The boxplots of above essential features in single-omics including gene expression, exon expression, and DNA methylation were generated by using the ggpubr package of R software (Jiang et al., 2018) to illustrate the distribution of above essential features in positive and negative groups. Since the features of somatic mutation are binary, they were annotated by the online tool of cBioPortal (Ricketts and Linehan, 2015).

### MPA Modeling for Colon Cancer

The MPA modeling requires three elements: (1) profiles of prognosis-related features, (2) samples with classification indicators and (3) appropriate machine learning methods. Here, 15 MPA models were created based on different combinations of descriptors including (1) four single-omics, which including 12, 39, 22, and 32 features, respectively, (2) six combinations of double-omics data, which including 51, 34, 44, 61, 71, and 54 features, respectively, (3) four combinations of triple-omics data, which including 93, 66, 83, and 73 features, respectively, and (4) combination of all quadruple-omics data, which including all 105 features. Further, training and testing datasets for the MPA model were obtained through the Diverse Subset sampling method (Yuan et al., 2017). Typically, the first sample A was randomly selected as the seed for the training dataset. Secondly, sample B with the farthest spatial distance toward sample A (in here, represents the spatial distance between omics profiles of two samples) was selected to put into the training dataset. Thirdly, the third sample with the farthest average distance from both samples A and B were extracted. Finally, sampling was repeated until two-thirds of the positive and negative samples were extracted as the training set, and the remaining samples were defined as the testing set. Here, the above essential features were taken as the potential prognosis-related features, of which the profile was set as the feature profiles of the model training and testing. For machine learning approaches, Support Vector Machines (SVM), Neural Network (NN), Naïve Bayes (NB), Logistic Regression (LR), Random Forest Classifier (RF), Linear Regression (LiR), Keras Depp Learning (Keras) were implemented by using the python 3.7 package of sklearn, TensorFlow and keras to generate the MPA model.

### Target Regulation Simulation (TRS) Process for Essential Features

**FIGURE 1:**
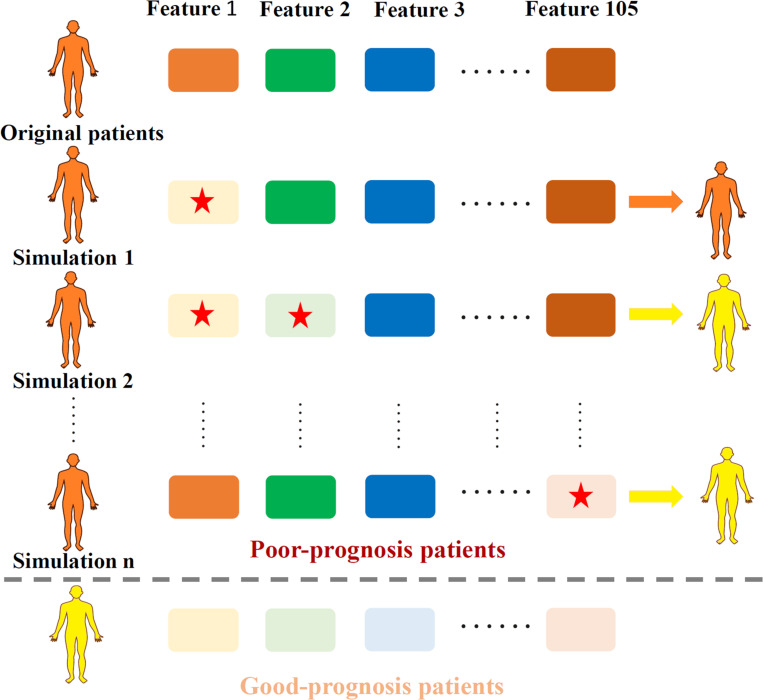
Illustration of the TRS process. Original samples with poor-prognosis were marked in orange while good-prognosis samples were marked in yellow. Each time, one or two features (marked with a red star) were down-regulated according to the expression level of good-prognosis samples, causing the original samples to be re-classified according to the MPA model.

To generate the TRS process, the profiles of total 105 essential features in quadruple-omics, as well as the corresponding OS in the overlapped TCGA samples of four omics were utilized to the MPA model training and testing. After model testing, all the TCGA samples in the testing set were pre-clustered as prognosis positive and negative ones according to the OS days of 1,825. For each true negative sample, the expression profile of 105 individual features and 5,460 two-feature combinations were retrieved for simulation. Each selected individual feature or features in combinations of the true negative samples were down-regulated to the minimum value of those in positive patients, then these samples were re-evaluated through the MPA model to obtain a new classification label. The process of TRS was illustrated in Figure 1.

### Survival Analysis of Samples

Survival analysis (Schlumberger et al., 2017) was performed based on the classification results of testing samples by the LR method. Then, Kaplan–Meier survival curves of different types of samples were evaluated using the survival and survminer packages of R software (Modhukur et al., 2018). Besides, the log-rank test (Katai et al., 2018) was employed to test the difference between the two compared sample groups.

### Protein–Protein Interaction (PPI) Network of Essential Biomarkers

Essential features were used to construct a PPI network. The essential features of each single-omics dataset derived above were transformed into gene symbols based on the annotation information downloaded from the UCSC Xena database. Further, gene symbols of the quadruple-omics datasets were integrated and annotated into the background PPI network using Cytoscape software version 3.4.0 (Kohl et al., 2011). Different colors were used to distinguish different omics types.

### Pathway Integration Analysis of Essential Features in the PPI Network

For essential features in the PPI network, relationship between the corresponding gene symbol and colon cancer was investigated using literature search and annotated into biological pathways, including KEGG version 87.0 (Kanehisa et al., 2017) and GeneCards version 4.12 (Rappaport et al., 2017). Pathway integration analysis was performed by Edraw max version 8.6 (Deng et al., 2019).

### Feature Comparison From Different Aspects

To evaluate the performance of features derived from different aspects, 33 features including 13 from the PPI network, 8 from the integrated pathway and 12 from the TRS process were analyzed and compared. All samples in the dataset were grouped by the median value of these individual features, respectively, and then survival analysis was performed to compare the potential PRBs from the above three different aspects. Further, all detected 13 PRBs from these three aspects were used to generate the Linear Regression model by sklearn package of python 3.7.

### Prognostic Evaluation of Samples With Different Clinical Information

To evaluate the clinical information of patients, 11 clinical features including age, gender, weight, histological type, history of colon polyps, person neoplasm cancer status, lymphatic invasion, pathologic stage, pathologic T stage, venous invasion and number of first degree relatives with cancer diagnosis were firstly evaluated through the Cox proportional hazard (PH) model. Then, the samples were classified by the above prognostic risk features. Here, 13 PRBs were individually evaluated to estimate the survival difference by setting the median value of each PRBs as the cutoff for prognostic classifications.

## Results

### Differential Expression Profiles of COAD Patient Based on Multi-Omics Analysis

**FIGURE 2:**
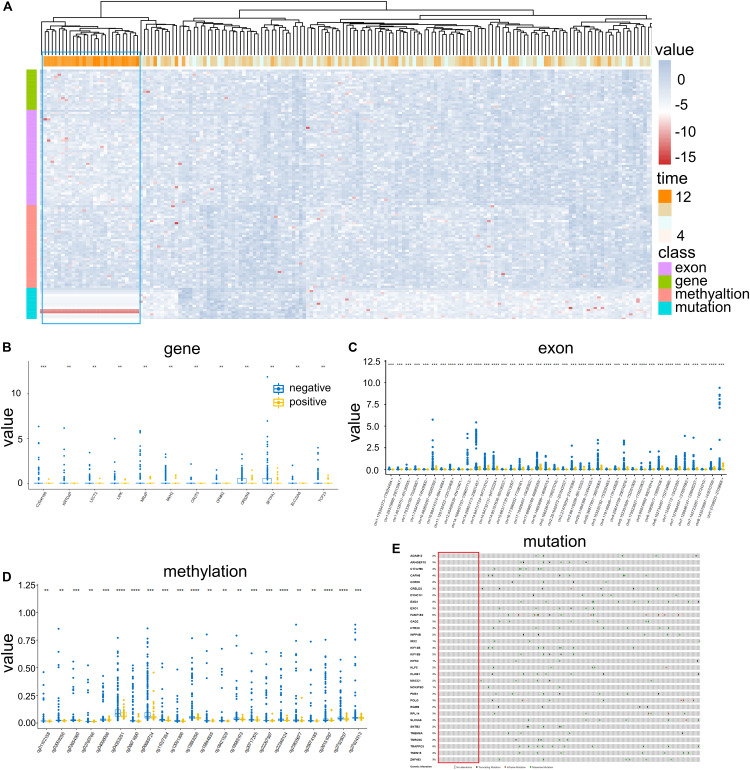
Differential expression profiles of COAD patients. **(A)** Clustering of 202 patient samples based on omics data corresponding to gene expression, exon expression, DNA methylation, and mutation. Each line shows one factor represented by corresponding features, while each column represents one patient sample. Each omics dataset and OS were logarithmically transformed for normalization. **(B)** Expression of 12 genes in positive and negative samples. **(C)** Expression of 39 exons in positive and negative samples. **(D)** Expression of 22 DNA methylation marks in positive and negative samples. **(E)** Mutation profiles of 32 genes in different patient samples. **P < 0.01 and ***P < 0.001.

To determine the essential features that closely related to the prognosis of COAD patients, differential expression features for four omics data were initially derived by setting appropriate conditions with *P* < 0.01 and FC > 1.5 or FC < 2/3 (see section “Materials and Methods”). Thus, 146 features for gene expression, 1,000 features for exon expression, 362 features for DNA methylation, and 968 features for somatic mutations were selected. After factor analysis, 19 factors for gene expression, 45 factors for exon expression, 39 factors for DNA methylation and 37 factors for mutations were determined. Thus, 140 factors were used to analyze patient samples based on quadruple-omics profiles (Figure 2). The expression profiles of 202 overlapped patient samples included in all quadruple-omics profiles are illustrated in Figure 2A, in which samples with high-OS were mostly clustered into one branch (marked with blue dotted box). In that case, the overall expression profiles contained all quadruple-omics data that could significantly distinguish high-OS and low-OS patients.

Moreover, by removing redundancy, 105 unique essential features including 12 for gene expression (Figure 2B), 39 for exon expression (Figure 2C), 22 for DNA methylation (Figure 2D) and 32 for somatic mutations (Figure 2E) were retained. The further analysis illustrated that the expression levels in negative groups were generally higher than those in positive ones for both gene expression, exon expression and DNA methylation. For somatic mutation, none of the positive samples contained alterations on those 32 essential features (Figure 2E), while truncating mutations, in-frame mutations and missense mutations frequently occurred in negative groups (Supplementary Table S2). Thus, all the above essential features could be considered as prognosis-related features that were differentially expressed between the positive and negative samples from TCGA.

### Performance of MPA Modeling

**FIGURE 3:**
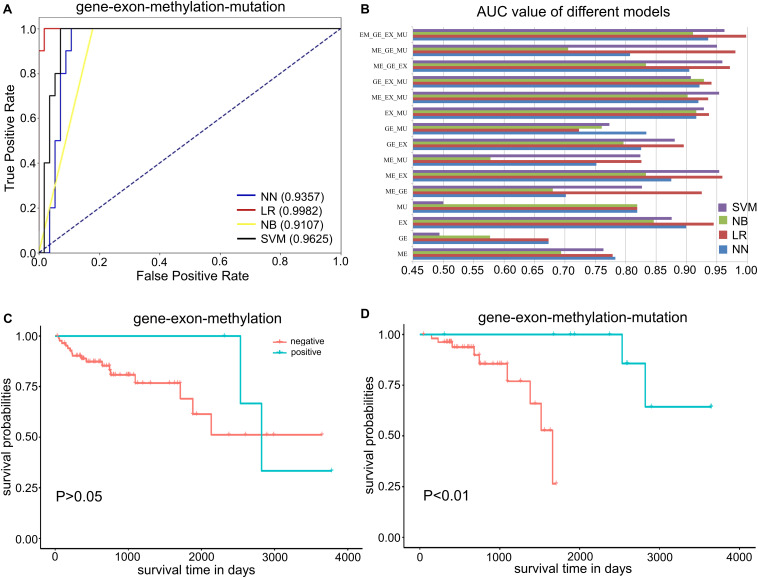
Performance of the MPA model for COAD prognostic analysis. **(A)** ROC curves of the quadruple-omics MPA model. Different machine learning approaches are represented by different lines. **(B)** AUC values of different MPA models, including single-omics data and combinations of multi-omics data. “GE,” “EX,” “ME,” “MU” stands for “gene,” “exon,” “methylation,” “mutation,” respectively. **(C)** Survival curves determined by the MPA model based on the combination of gene expression, exon expression, and DNA methylation. **(D)** Survival curves determined by the MPA model based on the combination of gene expression, exon expression, DNA methylation, and somatic mutations.

The MPA modeling was established based on 15 different combinations of omics profiles, and further been evaluated through four machine learning approaches including SVM, NN, NB, and LR for comparison. The receiver operating characteristic (ROC) curves of all 15 models were illustrated in Supplementary Figure S1. Results illustrated that the best MPA model for single-omics data could achieve the AUC value of 0.945 as the baseline, which could be further increased to 0.959 for the combination of double-omics data and 0.980 for triple-omics data. By integrating all four omics data, the classification performance could reach to the AUC of 0.998 for MPA modeling on LR (Figure 3A), followed by 0.963 for SVM, 0.936 for NN and 0.911 for NB (Figure 3B). Since the LR model revealed the best prediction performance among other approaches, it was chosen for MPA modeling and further prognosis analysis.

Further, we evaluated our MPA model through survival analysis based on the predictions of the LR model. The KM survival curves indicated that the combination of triple-omics data was still unable to ideally distinguish the high-OS and low-OS samples (Figure 3C), even though the prediction performance is distinguishable. Remarkably, the KM survival curves for positive and negative groups predicted by the MPA model, which consisted of all four omics data could be perfectly distinguishable (Figure 3D). In that case, the MPA model based on 105 essential features derived from four omics data could be used for prognostic analysis of COAD patients.

### PPI Network and Pathway Integration Analysis of Survival-Associated Omics Features

**FIGURE 4:**
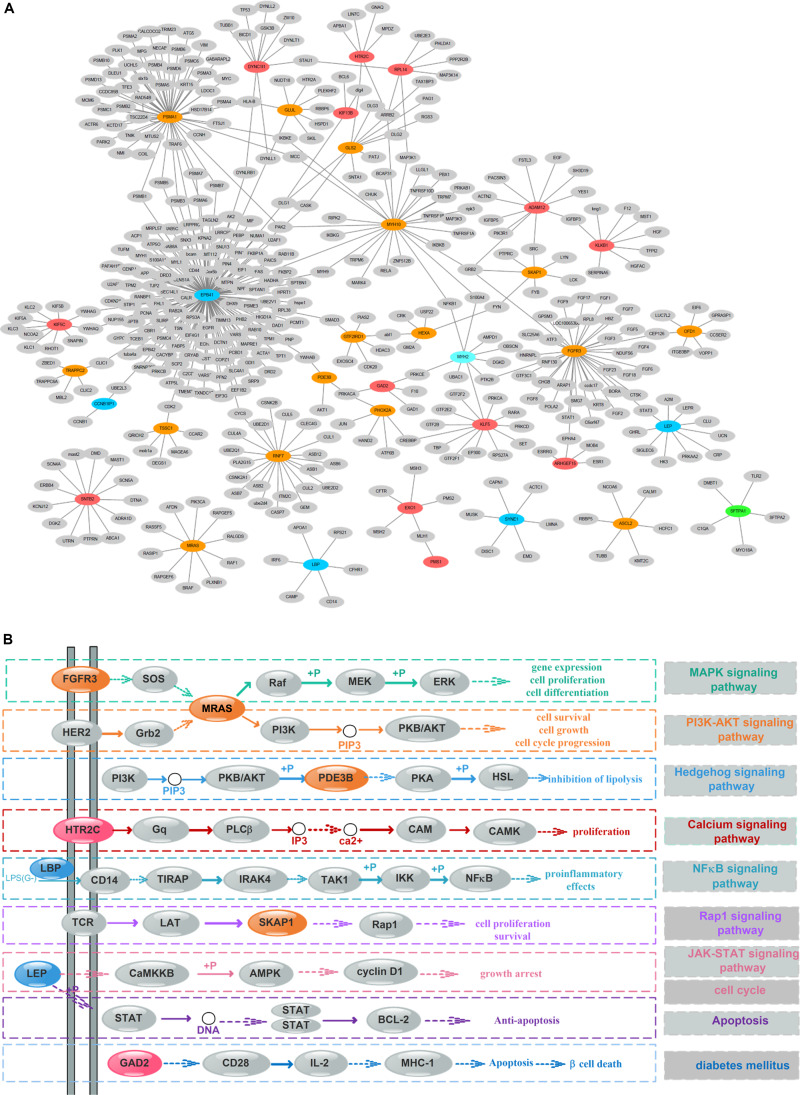
Protein–protein interaction (PPI) network and pathway involving the survival-associated features. **(A)** PPI network of 35 features; green, blue, orange, and red represent gene expression, exon expression, DNA methylation, and somatic mutations, respectively. Gray nodes represent the background. **(B)** Canonical cancer-related pathway in the KEGG database. The color of each node is the same as that in panel **(A)**.

To detect the PRBs, all prognosis-related features were transformed into non-redundant gene symbols including 12 for gene expression, 31 for exon expression, 26 for DNA methylation and 32 for somatic mutations (Supplementary Table S3). Since ZNF493 and MYH2 could be transformed from multi-omics data, 99 unique gene symbols were obtained (Supplementary Table S4). Among them, 45 genes could be annotated into an integrated PPI network. In particular, 30 genes were defined as hub nodes with a degree over or equal to 5, which indicates those genes might participate in crucial biological functions. Further analysis showed that all 45 genes mapped in the PPI network were associated with biological processes or pathways related to the pathogenesis and development of cancer (Table 1). The refined PPI network which contains 30 genes with the degree over 5 were illustrated in Figure 4A, and canonical cancer-related pathways of those features such as cell signaling pathways, cell cycle, apoptosis, and diabetes pathways which derived from KEGG database were illustrated in Figure 4B.

**TABLE 1:** The information of genes in the integrated PPI network.

Gene	Class	Color	Degree	Pathway	Database	PMID
GAD2*^#^	Mutation	Red	4	Type I diabetes mellitus*	KEGG	3003660^$^
KIF5C	Mutation	Red	10	Dopaminergic synapse	KEGG	18413843^$^
CCNB1IP1	Exon	Blue	2	Cell cycle	PubMed	19437480
OFD1	Methylation	Orange	7	Cell cycle	Genecards	20835237
KIF13B	Mutation	Red	3	∼	∼	23142292
GTF2IRD1	Methylation	Orange	6	cGMP-PKG signaling pathway	KEGG	23804703^$^
PDE3B*	Methylation	Orange	3	Hedgehog signaling pathway*	KEGG	24282571
RPL14	Mutation	Red	7	Ribosome	KEGG	24824907^$^
KLF5	Mutation	red	13	Glucose/energy metabolism	Genecards	25037223
LEP*	Exon	Blue	10	JAK-STAT signaling pathway, cell cycle, apoptosis*	KEGG	25250132
HTR2C*	Mutation	Red	6	Calcium signaling pathway*	KEGG	25323578^$^
FGFR3*	Methylation	Orange	37	MAPK signaling pathway*	KEGG	25623536
HEXA	Methylation	Orange	5	Metabolic pathways	Genecards	26401073
EXO1	Mutation	Red	5	Mismatch repair	KEGG	26714599
CRELD2^#^	Mutation	Red	2	∼	∼	27392906
KLKB1	Mutation	Red	8	Complement and coagulation cascades	KEGG	27779706^$^
LBP*	Exon	Blue	6	NF-kappa B signaling pathway*	KEGG	27876571
GLS2	Methylation	Orange	10	Alanine, aspartate and glutamate metabolism	KEGG	28007957
SYNE1	Exon	Blue	6	Cell cycle	Genecards	28058013
EPB41	Exon	Blue	128	Cell cycle	Genecards	28193906^$^
DYNC1I1	Mutation	Red	10	Cell cycle	Genecards	28193906^$^
GLUL	Methylation	Orange	8	Alanine, aspartate and glutamate metabolism	KEGG	28207045^$^
ARHGEF15	Mutation	Red	4	GPCR Pathway	Genecards	29073728
CAPN6^#^	Mutation	Red	2	Apoptosis Pathway	Genecards	29202800^$^
MRAS*	Methylation	Orange	10	MAPK/PI3K-AKT signaling pathway*	KEGG	29305742^$^
PSMA1	Methylation	Orange	54	wnt pathway	Genecards	29423100
ADAM12	Mutation	Red	11	Insulin growth factor-related pathway	PubMed	29731694
ASCL2	Methylation	Orange	6	∼	∼	29886802
POLG^#^	Mutation	Red	2	Metabolic pathways	Genecards	30002826
SKAP1*	Methylation	Orange	7	Rap1 signaling pathway*	KEGG	30183087^$^
TSSC1	Methylation	Orange	6	∼	∼	30231249
DAP^#^	Methylation	Orange	1	Apoptosis and autophagy	Genecards	30552554
MYH10	Methylation	Orange	28	∼	∼	∼
RNF7	Methylation	Orange	21	∼	∼	∼
SNTB2	Mutation	Red	13	∼	∼	∼
MYH2	Gene_exon	Lightblue	7	∼	∼	∼
PHOX2A	Methylation	Orange	5	∼	∼	∼
SFTPA1	Gene	Green	5	∼	∼	∼
TRAPPC2	Methylation	Orange	5	∼	∼	∼
SLC30A3^#^	Exon	Blue	3	∼	∼	∼
EXD3^#^	Mutation	Red	2	∼	∼	∼
FAM71E2^#^	Mutation	Red	1	∼	∼	∼
OPN4^#^	Exon	Blue	1	∼	∼	∼
PMS1	Mutation	Red	1	∼	∼	∼
PPP4R4^#^	Exon	Blue	1	∼	∼	∼

^*a*^Columns 1–4 stands for the gene name, corresponding omics data type, color and degree of genes illustrated in the PPI network, respectively. Columns 5 and 6 represent the pathway name and database of gene annotations. Column 7 represents the PMID of the corresponding literature. * Genes and pathways involved in the integrated pathway in Figure 4B. The first column represents all genes that could be mapped to the PPI network. ^#^Genes not illustrated in Figure 4. ^$^Pathway in which the gene located is associated with colon cancer.

Previous researches reported that signaling pathways are frequently altered in cancers (Sanchez-Vega et al., 2018). Here, FGFR3 and MRAS in the MAPK signaling pathway were found to participate in gene expression and cellular processes including cell proliferation and cell differentiation. Additionally, MRAS is involved in the PI3K-AKT signaling pathway related to cell survival, cell growth, and cell cycle progression, while PDE3B is found in the Hedgehog signaling pathway, which is related to the inhibition of lipolysis. Besides, HTR2C which participated in the calcium signaling pathway is potentially associated with cell proliferation. Moreover, LBP involved in the NF-κB signaling pathway is associated with pro-inflammatory effects, and SKAP1 in the Rap1 signaling pathway is involved in cell proliferation and survival.

Besides, genetic alterations that control cell cycle progression and apoptosis are considered to be common hallmarks of multiple cancer types (Sanchez-Vega et al., 2018). LEP is found to be involved in the cell cycle, JAK-STAT signaling pathway and apoptosis to regulate tumor growth arrest and apoptosis. Moreover, emerging evidence from observational studies and meta-analyses suggest that diabetes mellitus is associated with an increased risk of cancer, as well as cancer incidence or prognosis (Noto, 2018). In particular, diabetes has been validated as a prognostic factor in stages I to III colorectal cancer patients (Croft et al., 2018), in which GAD2 involved could induce β-cell death. Other cancer-associated processes or pathways and literature evidence for the occurrence or development of colon cancer can be found in Table 1. Thus, features such as EPB41, PSMA1, FGFR3, MRAS, and LEP which were involved in cancer-related pathways and with high PPI degrees (>/ = 10) were considered as PRBs for further analysis.

### Detection and Evaluation of Prognosis Risk Biomarkers for COAD

**FIGURE 5:**
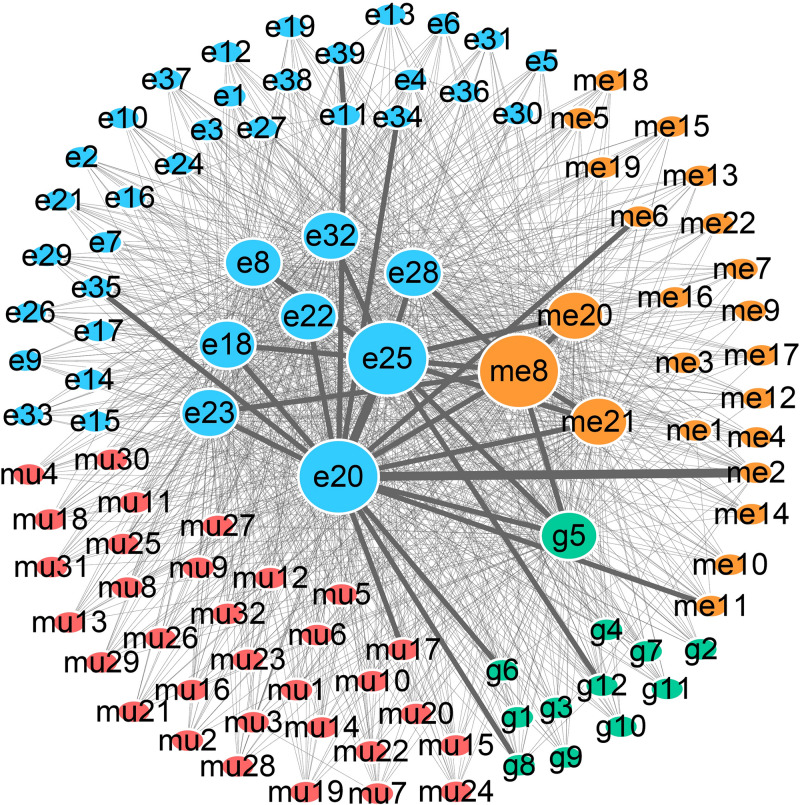
Network of prognosis-related features that could change the prognosis of COAD patients. Green, blue, orange, and red nodes represent gene expression, exon expression, DNA methylation, and somatic mutations, respectively. The size of each node represents the number of patients that are affected by the corresponding features. Lines linking two nodes indicate that the combination of the two nodes can change the prognosis of patients, and the number is represented by the thickness of each line.

To further explore how the above prognosis-related features affect the prognosis of COAD patients, we performed *in silico* TRS modeling (see section “Materials and Methods”). The expression value of features in the negative group (low-OS patients) was individually or assembly adjusted to normal levels as those in the positive group (high-OS patients). Then, the prognosis level of adjusted patients was simulated through the MPA model. Here, 105 single prognosis-related features and 5,460 combinations of two features were systemically adjusted for simulation. Results showed that 12 out of 105 single features and 1,210 out of 5,460 combined features could change the prognosis of TCGA patients. As been illustrated in Figure 5, each node represents 1 of 105 single prognosis-related features and each line links two nodes represents one feature pair. Detailed information of nodes can be found in Supplementary Table S5. Since the regulation of single or combined prognosis-related features could change the prognosis of TCGA patients, it is possible to detect the PRBs for COAD, and thus, drugs that targeting the corresponding PRBs might be helpful for COAD patients. It can be found that the most essential nodes include me8 (cg06685724), e20 (chr2:106226785–106227016:−), e25 (chr20:36977951–36978065:+) representing C7orf46, LOC285000 and LBP, respectively. More importantly, the above three features were involved in 104 out of 1,210 feature combinations, which exceeding other features. In that case, C7orf46, LOC285000, and LBP were defined as essential prognosis biomarkers detected by TRS modeling.

Finally, we evaluate the performance of features derived from different aspects including 13 from the PPI network, 8 from the integrated pathway and 12 from the TRS modeling. The survival analysis was performed for each feature based on the median value as the classification indicator to evaluate the performance. Results showed that the 4 features including ZNF35 (cg20717205), LOC285000 (chr2:106226785–106227016:−), SLC30A3 (chr2:27479254–27479388:−) and LECT2 derived from TRS modeling could distinguish patients with different OS (Supplementary Figure S2). For PPI, 3 features that hold the potential to distinguish patients with different OS were detected, including LEP (chr7:127894457–127897682), RNF7 (cg06671690) and DYNC1I1 (Supplementary Figure S2). Among them, LEP was also involved in the JAK-STAT signaling pathway.

Thus, through PPI network and pathway integration analysis of all prognosis-related features detected by MPA modeling, 7 essential PRBs including EPB41, PSMA1, FGFR3, MRAS, LEP, RNF7, and DYNC1I1 were identified. Further, by combining with TRS modeling, C7orf46, LOC285000, LBP, ZNF35, SLC30A3, and LECT2 were also added and a total of 13 PRBs were eventually detected by integrating MPA and TRS modeling. Further, a prognosis risk scoring (PRS) model based on above 13 PRBs were constructed to evaluate whether those markers could distinguish high-OS and low-OS patients. By using linear regression, the PRS could be described based on the following equation (1):

(1) $$\begin{array}{c} \text{PRS}=\left(-1.021^{*}EPB41\right)+\left(-0.364^{*}PSMA1\right)+\\ \left(-0.046^{*}FGFR3\right)+\left(-0.113^{*}MRAS\right)+\left(-0.013^{*}LEP\right)+\\ \left(-0.219^{*}C7orf46\right)+\left(-0.379^{*}LOC285000\right)+\\ \left(-0.085^{*}LBP\right)+(-0.539^{*}ZNF35)+\\ (-0.150^{*}SLC30A3)+(-0.057^{*}LECT2)+\\ (-0.404^{*}RNF7)+(-0.143^{*}DYNC1I1)+0.325 \end{array}$$

The performance of the above PRS model could reach to 0.825 for AUC value, and by setting the best threshold of 0.254, the sensitivity of 0.900 and specificity of 0.625 could be achieved.

### Validation of PRBs on Independent Testing Dataset

**FIGURE 6:**
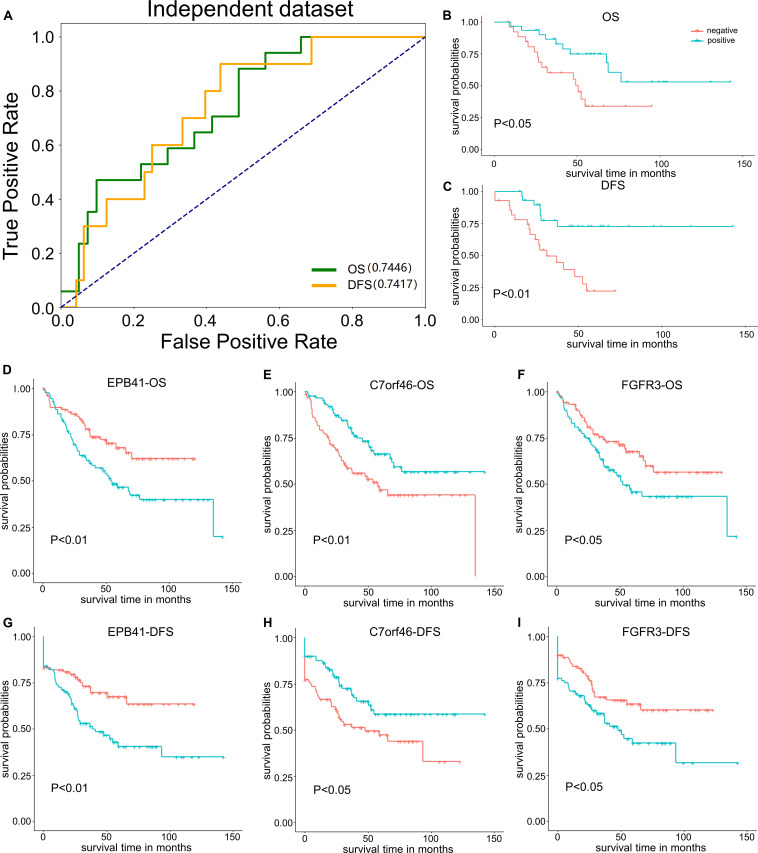
Performance of PRB gene signatures on independent testing dataset. **(A)** AUC value of 13 PRBs on independent testing dataset by set OS and DFS as classification indicators. **(B)** Survival analysis of 13 PRBs based on OS. **(C)** Survival analysis of 13 PRBs based on DFS. **(D)** Survival analysis of EPB41 based on OS. **(E)** Survival analysis of C7orf46 based on OS. **(F)** Survival analysis of FGFR3 based on OS. **(G)** Survival analysis of EPB41 based on DFS. **(H)** Survival analysis of C7orf46 based on DFS. **(I)** Survival analysis of FGFR3 based on DFS.

To evaluate the practicability and scalability of the PRBs mentioned above, 13 PRBs explored in this study were used to validate the performance in independent datasets downloaded from the NCBI GEO dataset (see section “Materials and Methods”), which included 177 patients with survival information from Moffitt Cancer Center. Here, all potential PRBs detected above were first translated into gene signatures. Then, the expression profiles of the above gene signatures were used to generate the classification model for patient samples by setting the cutoff of OS and disease-free survival (DFS) as 5 years. Results indicated that the prediction model constructed on the above gene signatures could achieve the AUC value of 0.745 for OS and 0.742 for DFS, respectively (Figure 6A). Moreover, the survival analysis based on the prediction of the gene signature-based model indicated that by integrating all the above features, the classification results could successfully distinguish the positive and negative samples for both OS (Figure 6B) and DFS (Figure 6C). Furthermore, by setting the median expression value as the cutoff in the independent testing dataset, gene signatures of EPB41, C7orf46, and FGFR3 could also distinguish the positive and negative samples classified by both OS and DFS, has been illustrated in Figures 6D–6I.

Moreover, the single cell-based expression dataset of colon cancer was obtained from the NCBI GEO database with the accession number of GSE18161 (Li et al., 2017), which included the count and FPKM of tumor/NM cells, as well as the count and FPKM of tumor/NM epithelial cells. Further, the Wilcoxon Test and Fold change were used to evaluate whether the above PRBs were differentially expressed between tumor and normal cells (Supplementary Table S6). Results showed that gene signatures such as PSMA1, FGFR3, C7orf46, RNF7, and ZNF35 were differentially expressed in normal and tumor samples with the *P*-value < 0.05. Other gene signatures including EPB41, MRAS, LEP, LOC285000, LBP, LECT2, SLC30A3, and DYNC1T1 were also differentially expressed in normal and tumor samples with |FC|> 2 (Supplementary Figure S3). Thus, the evaluation of the corresponding gene signatures for PRBs through the independent testing dataset illustrated that above 13 PRBs detected by MPA and TRS modeling could be defined as classification indicators to predict the prognosis of COAD patients.

### PRBs Illustrated Different Affections for Patients With Different Conditions

It should be noticed that clinical information which reflect different conditions of patient samples might affect the prognosis of colon cancer (Vergo and Benson, 2012; Dienstmann et al., 2015; Karvinen and Vallance, 2015). Thus, 11 personalized clinical features including age, gender, weight, histological type, history of colon polyps, person neoplasm cancer status, lymphatic invasion, pathologic stage, pathologic T stage, venous invasion and number of first degree relatives with cancer diagnosis, which may closely related with cancer prognosis were selected to the prognostic risk assessment with Cox PH regression model. Results showed that age, weight, person neoplasm cancer status, lymphatic invasion, pathologic stage, pathologic T stage, and venous invasion were detected as the colon cancer prognosis-related risk factors (Supplementary Table S7). Further, samples in our testing dataset were grouped by personalized prognostic risk features as well as the number of first degree relatives with cancer diagnosis. For example, for the risk factor of age, all patients were separated into two groups, age over 65 or age less than or equal to 65. Then, all 13 PRB-related gene signatures were individually analyzed by setting the median value as the cutoff to classify the prognosis difference in each group through survival analysis.

The co-occurrence and exclusivity of PRBs could be detected in patients with different personal conditions. For example, SLC30A3 could significantly distinguish positive and negative samples for both patients older than 65 or younger than 65. However, PRBs such as LOC285000 and LEP could only distinguish those patients older than 65, while DYNC1I1 only illustrate significance in patients younger than 65 (Supplementary Figure S4). For person neoplasm cancer status, LOC285000 and LEP could distinguish the prognosis of patients free from neoplasm, while SLC30A3 and DYNC1I1 were significant for patients with neoplasm (Supplementary Figure S5). Thus, PRBs illustrated different affections for patients with different personalized clinical conditions which were closely associated with the prognosis of COAD. Detailed information of different sample groups classified by personalized prognostic risk features (Supplementary Table S8) and the corresponding significant PRBs (Supplementary Table S9) were provided.

## Discussion

Identification of PRBs in colon cancer is essential for the diagnosis, monitoring, and treatment of patients. By taking advantage of next-generation sequencing technologies, large-scale data could be obtained for *in silico* analysis to reveal PRBs. Here, we presented the MPA and TRS modeling to detect the PRBs for COAD. First, by integrating multi-omics data from gene expression, exon expression, DNA methylation, and somatic mutations. Then, features selection were obtained through dimensionality reduction based on factor analysis. After that, all 105 essential features from quadruple-omics data were integrated to generate the MPA model. Furthermore, 45 prognosis-related features were obtained through the analysis of PPI networks and mapped into multiple cancer-related pathways. Among them, some prognosis-related features were directly related with the occurrence, development or prognosis of cancer, such as PSMA1 (degree = 54) was identified as colon cancer markers by proteomic profiling (Yang et al., 2018), FGFR3 (degree = 37) was related with multi-regional colon cancer through inter- and intra-tumor profiling (Kogita et al., 2015), ALPi is selectively induced by HDACi in colon cancer cells in a KLF5 (degree = 13) dependent manner (Shin et al., 2014), GLS2 (degree = 10) was validated as differential expression gene in colon cancer cells (Alix-Panabieres et al., 2017) and LEP (degree = 10) was examined to be associated with the development of colorectal cancer (Rezaei-Tavirani et al., 2013). Furthermore, several pathways were provided to be colon cancer-related pathways, such as wnt pathway, PI3K-AKT signaling pathway and cell cycle signaling pathway were reported as common oncogenic signaling pathways (Sanchez-Vega et al., 2018), which could be regulated by PSMA1 (degree = 54), MRAS (degree = 10), and LEP (degree = 10), respectively. Detailed information of all prognosis-related features including degrees, corresponding pathways, and literature evidence was listed in Table 1.

Elaborate investigation of TCGA samples indicated that the above prognosis-related features were mostly overexpressed in negative samples. To further illustrate the therapeutic actionability, TRS modeling was processed to detect potential targets for inhibitors. Generally, TRS could simulate the prognosis classifications of TCGA patients with expression levels altered for individual or combined prognosis-related features. After scanning all single and combinations among 105 prognosis-related features in low-OS patients, results indicated that alter the expression level of features such as chr20:36977951–36978065:+ (LBP), chr2:106226785–106227016:− (LOC285000), and cg06685724 (C7orf46), the classification could be switched from low-OS to high-OS. Thus, by integrating MPA and TRS modeling, 8 features including chr1:29315868–29315947:+ (EPB41), cg02654360 (PSMA1), cg23835677 (FGFR3), cg18421529 (MRAS), chr7:127894457–127897682:+ (LEP), cg06685724 (C7orf46), chr2:106226785–106227016:− (LOC285000) and chr20:36977951–36978065:+ (LBP) were detected as potential PRBs. Among them, LOC28500 and LBP were also been identified as prognostic risk factors by both univariate and multivariate analysis of the Cox PH regression model (Supplementary Table S10). Moreover, FGFR3 has already been proved to be an essential drug target for multiple cancer types. For example, XL999, which targeting FGFR3, has the potential to prevent tumor growth and has been investigated for the treatment of unspecified cancer/tumors (Supplementary Table S11). In addition, to compare the prognostic performance of the prognosis-related features of colon cancer from different criteria, we evaluated the performance of the features derived from the PPI network, integrated pathway, and TRS modeling. The results of survival analysis showed that features extracted from TRS modeling reflected better performance by comparing with those derived from the PPI network and integrated pathway, indicating that the TRS modeling might be an efficient strategy to explore PRBs for cancer prognosis. By combining 13 PRBs from different aspects, the linear regression model could reach an AUC value of 0.825. Thus, the strategy of screening PRBs from different aspects might better reflect the prognostic features of cancer patients as previous studies reported (Chen S. et al., 2019; Chen Y. H. et al., 2019; Wang et al., 2019).

Also, the above 13 PRBs were also reported to affect the progression and prognosis of different cancers. For example, EPB41, PSMA1, LEP, LECT2, and ZNF35 were associated with breast cancer (Kao et al., 2005; Deng et al., 2006; Andres et al., 2015; Feng et al., 2019). PSMA1, MRAS, LEP, SLC30A3, and RNF7 were found closely related with prostate cancer (Singh et al., 2016; Sun et al., 2016; Wang et al., 2016; Xiao et al., 2017; Zhu et al., 2017). FGFR3, LBP, LECT2, and DYNC1I1 were distinguishable markers in lung cancer (Wang et al., 2017, 2018; Zhang Y. et al., 2017; Hung et al., 2018). Besides intra- validation, the generality and repeatability of PRBs were evaluated through an independent dataset from the GEO database. The results of both classification and survival analysis indicated that the PRBs and corresponding gene signatures determined here could effectively distinguish the samples with different prognostic independent dataset, which could be used as prognostic classification indicator for COAD patients.

Furthermore, it is noted that among the above 13 PRBs, five PRBs of chr1:29315868–29315947:+ (EPB41), chr7:127894457–127897682:+ (LEP), chr2:106226785–106227016:− (LOC285000) chr20:36977951–36978065:+ (LBP), and chr2:27479254–27479388:− (SLC30A3), were from exon expression, while other six PRBs including cg02654360 (PSMA1), cg23835677 (FGFR3), cg18421529 (MRAS), cg06685724 (C7orf46), cg20717205 (ZNF35), and cg06671690 (RNF7) were from DNA methylation. And one of the PRBs LECT2 was from gene expression, and the left one DYNC1I1 was from somatic mutation. This means the expression level of exon expression and DNA methylation might be more important for the prognosis of COAD patients rather than gene expression and somatic mutations. Meanwhile, by integrating quadruple-omics data with appropriate machine learning approaches such as logistic regression, the prognosis prediction performance could be further increased to 0.998 based on 105 essential features. Thus, with the accumulation of multi-omics data and improvement of machine learning approaches, the PRBs for multiple cancer types could be detected and accelerate the development of cancer therapeutics.

## Conclusion

In this paper, we constructed the MPA model to comprehensively reveal the PRBs for COAD patients based on gene expression, exon expression, DNA methylation, and somatic mutations. Besides the high performance of the MPA model for prognostic classification, 105 essential features that were closely related to COAD prognosis were detected. Furthermore, by screening through the criteria of the PPI network, cancer-related pathway and TRS modeling, essential features with gene symbols of EPB41, PSMA1, FGFR3, MRAS, LEP, C7orf46, LOC285000, LBP, ZNF35, SLC30A3, LECT2, RNF7, and DYNC1I1 were identified as PRBs for COAD patients. In addition, evaluation of the independent testing dataset and single-cell based RNA-seq dataset illustrated the PRBs and corresponding gene symbols detected in this study could successfully distinguish COAD patients with different prognosis. Finally, some of the PRBs were demonstrated to hold the potential to distinguish different prognosis in patients with different clinical conditions. The MPA and TRS modeling, as well as the PPI network and integrated pathway analysis presented here could only detect the PRBs to predict the prognosis of COAD patients, but also provide new perspectives for novel drug development and therapeutic applications for COAD treatment.

## Data Availability Statement

The raw omics and phenotypic data of COAD were obtained from the TCGA module of Public Xena Hubs in the UCSC Xena database.

## Author Contributions

TQ and ZY designed the study and wrote the manuscript. ZY collected the corresponding datasets and completed *in silico* analyses. XY and QW assisted in model construction. ZD and KT assisted in model validation. TQ and ZC supervised the whole project and edited the manuscript.

## Conflict of Interest

The authors declare that the research was conducted in the absence of any commercial or financial relationships that could be construed as a potential conflict of interest.
