# Supplementary material for: Detecting Prognosis Risk Biomarkers for Colon Cancer Through Multi-Omics-Based Prognostic Analysis and Target Regulation Simulation Modeling
Source: Front Genet. 2020 May 26;11:524. doi: 10.3389/fgene.2020.00524 (PMC7264416; doi:10.3389/fgene.2020.00524)
Supplement: Supplementary file 1 [file Data_Sheet_1.docx]

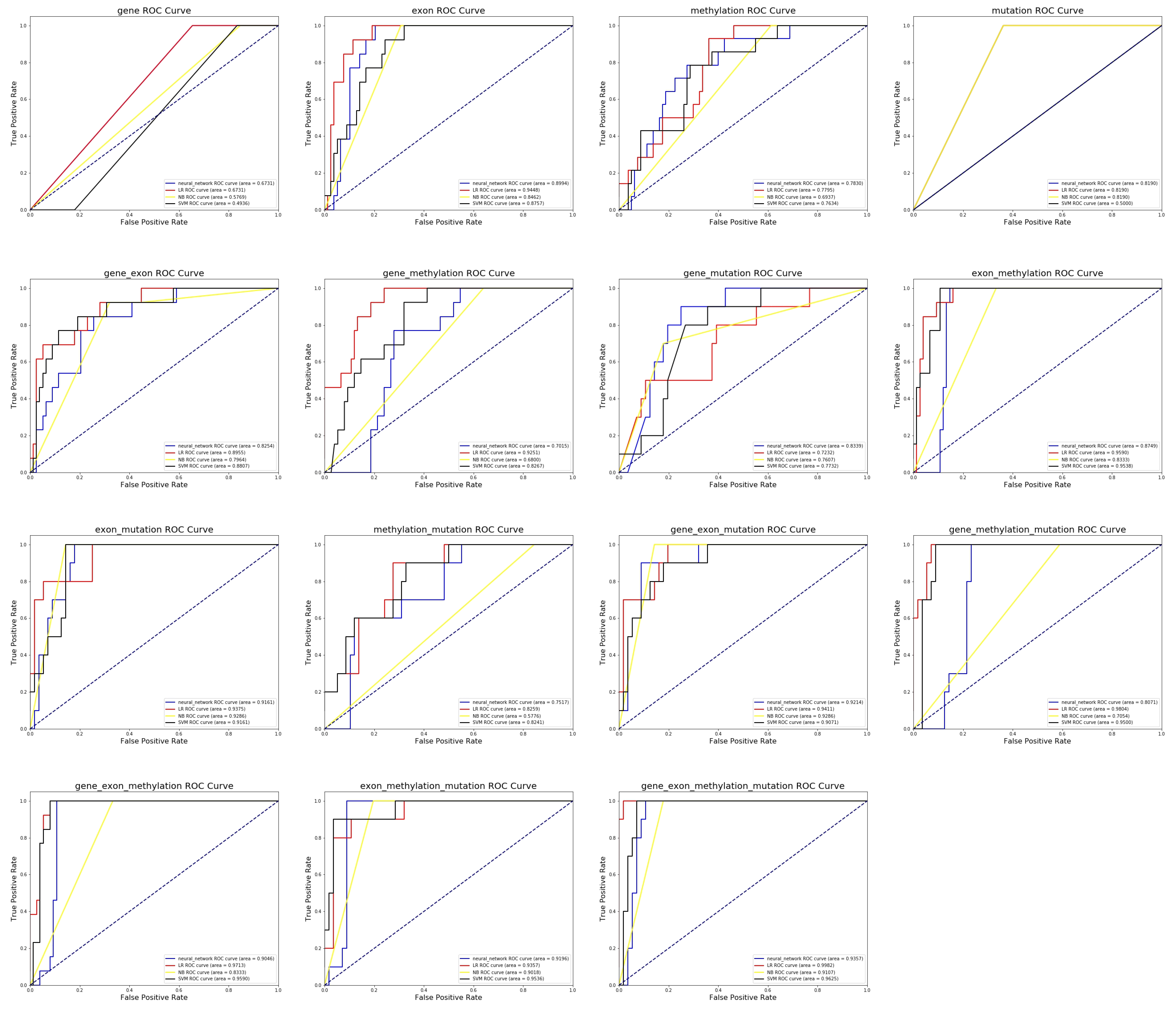


**Supplementary Figure 1. ROC curves of different MPA models based on single-omics, double-omics, triple-omics and quadruple-omics data.** Different machine learning approaches were represented by lines with different colors.


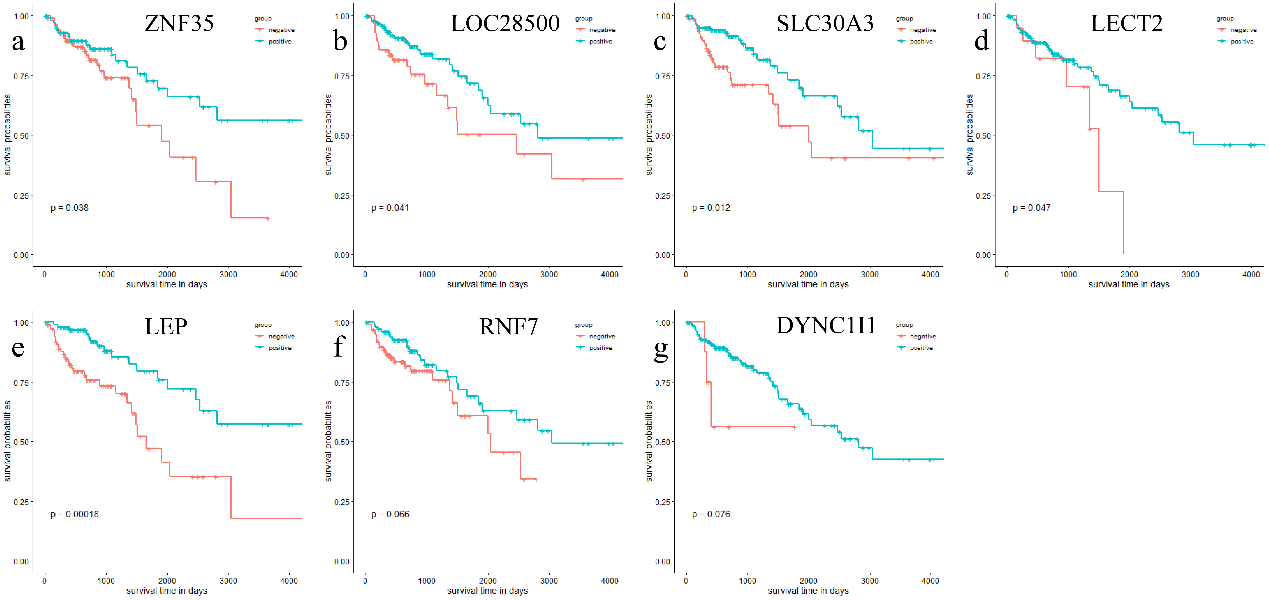


**Supplementary Figure 2. Survival curves for individual features detected from different criteria. (a)** Survival curve of ZNF35 (cg20717205). **(b)** Survival curve of LOC28500 (chr2:106226785-106227016:-). **(c)** Survival curve of SLC30A3 (chr2:27479254-27479388:-). **(d)** Survival curve of LECT2. **(e)** Survival curve of LEP. (f) Survival curve of RNF7. **(g)** Survival curve of DYNC1I1.


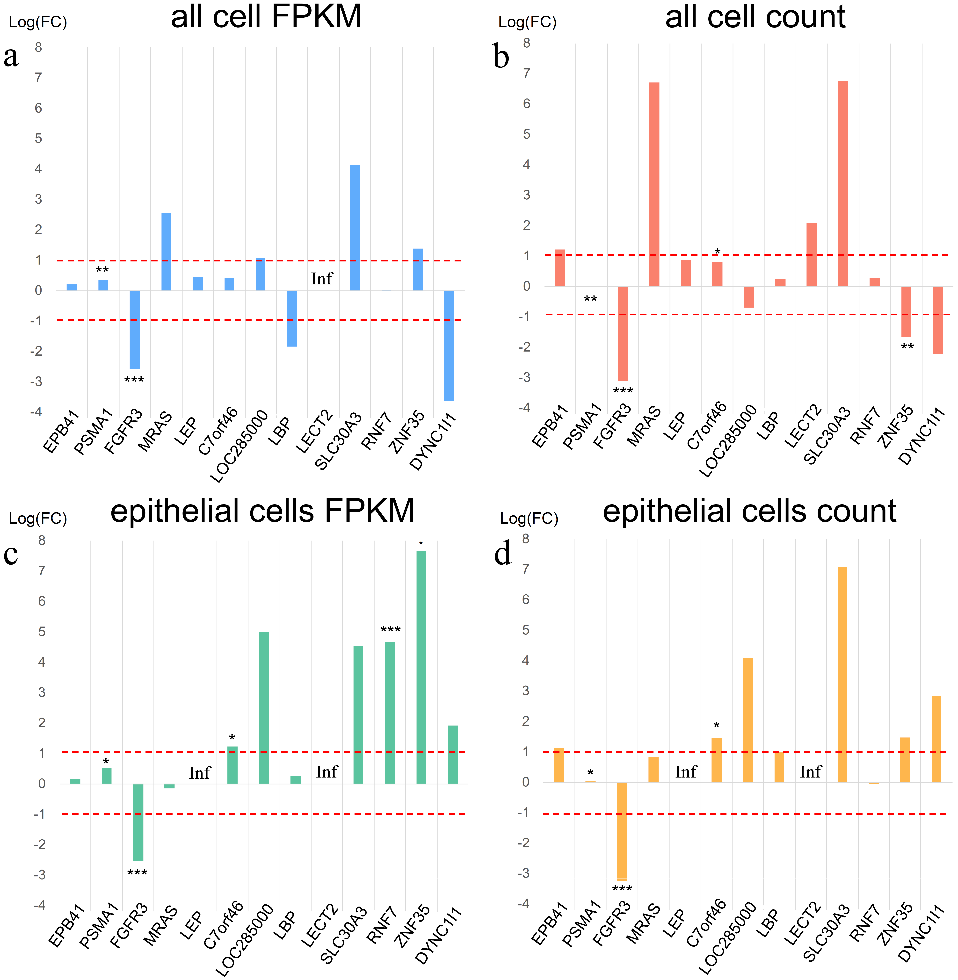


**Supplementary Figure 3. Fold change of PRBs between tumor cells and normal ones. (a)** Fold change of 13 PRBs on all cell FPKM. **(b)** Fold change of 13 PRBs on all cell count. **(c)** Fold change of 13 PRBs on epithelial cells FPKM. **(d)** Fold change of 13 PRBs on epithelial cells count. In each subgraph, stars represent the significance level of the Wilcoxon test. Inf means the denominator (expression in normal samples) is zero.


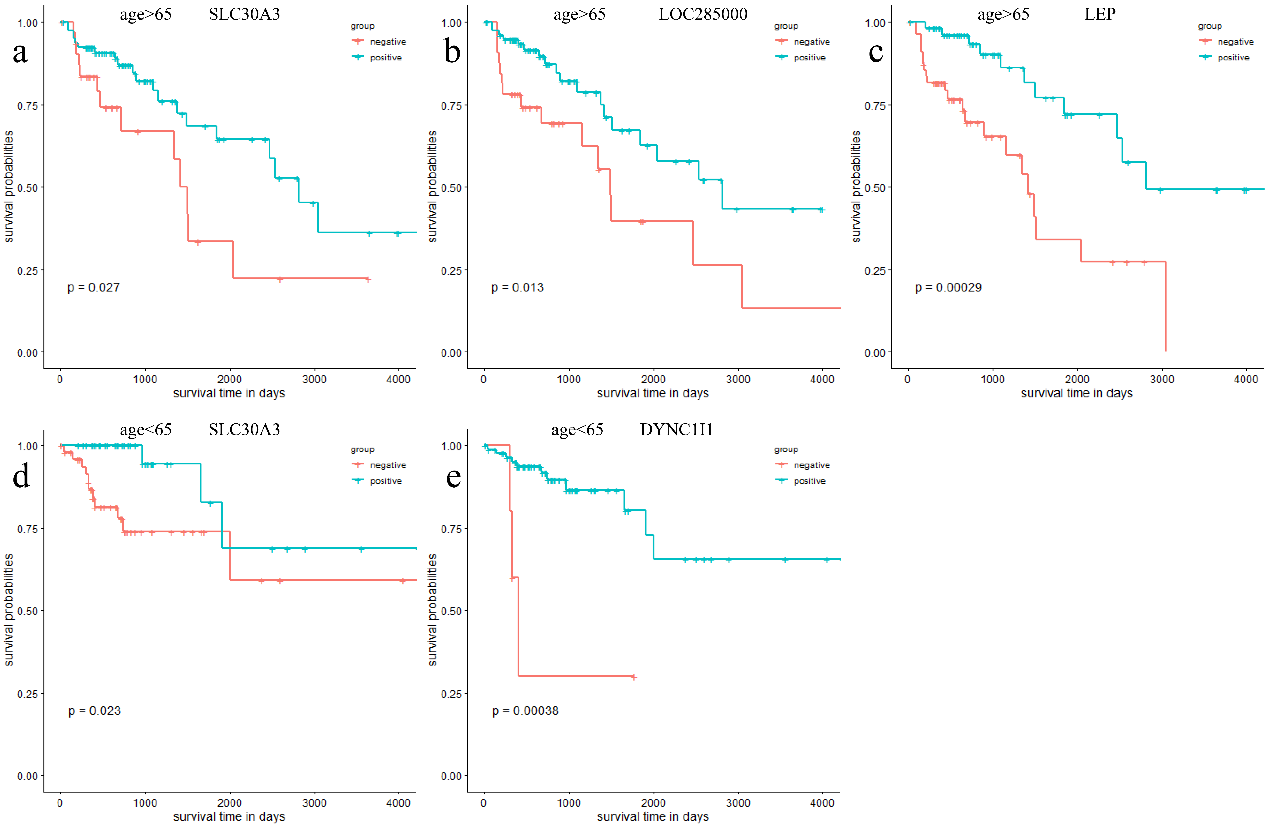


**Supplementary Figure 4. Survival analysis of significant PRBs in different age groups.** **(a)** Survival analysis of SLC30A3 in group of patients over 65. **(b)** Survival analysis of LOC285000 in group of patients over 65. **(c)** Survival analysis of LEP in group of patients over 65. **(d)** Survival analysis of SLC30A3 in group of patients less than 65. **(e)** Survival analysis of DYNC1I1 in group of patients less than 65.


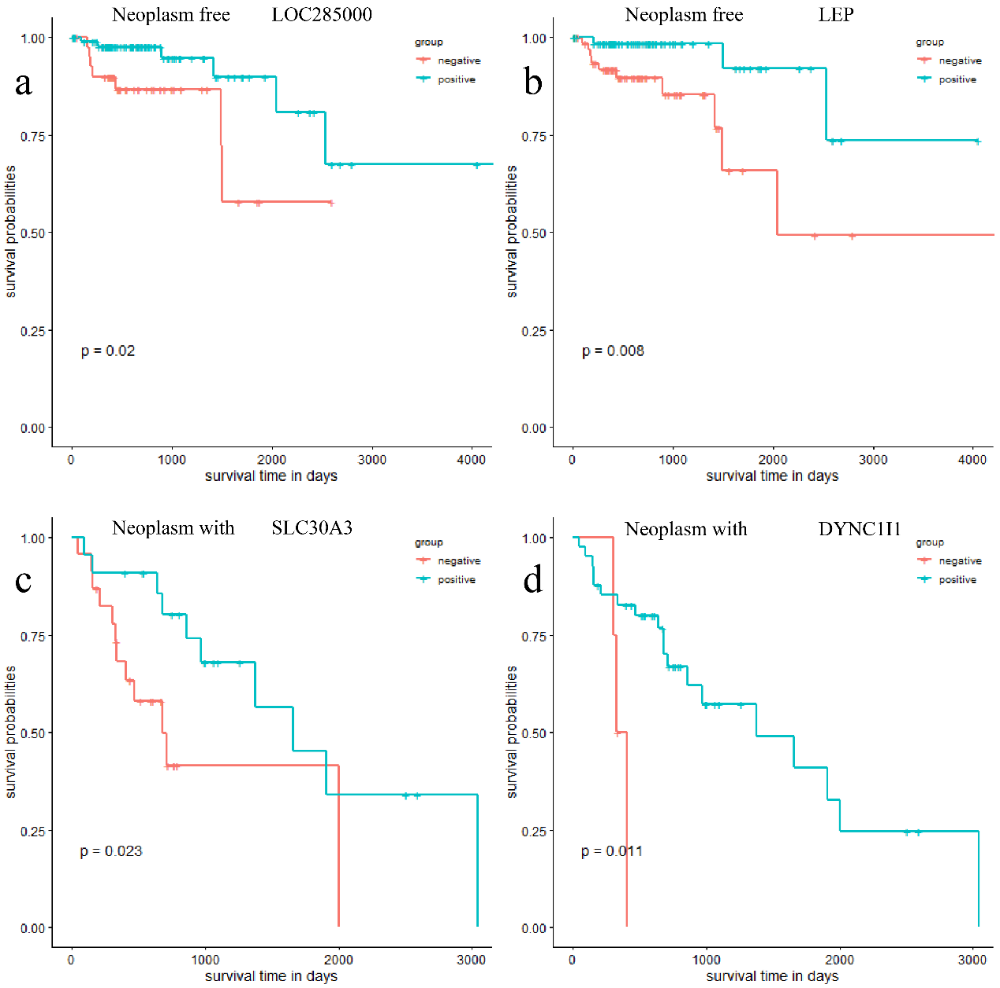


**Supplementary Figure 5. Survival analysis of significant PRBs in different neoplasm groups. (a)** Survival analysis of LCO285000 of patients with neoplasm free. **(b)** Survival analysis of LEP in group of patients with neoplasm free. **(c)** Survival analysis of SLC30A in group of patients with neoplasm. **(d)** Survival analysis of DYNC1I1 in group of patients with neoplasm.

**Supplementary Table 1. Overlapped TCGA samples for four omics data**

**Supplementary Table 1a. TCGA patients samples for gene expression ^a^.**

| ***sample*** | ***OS*** | ***sample*** | ***OS*** | ***sample*** | ***OS*** |
| --- | --- | --- | --- | --- | --- |
| TCGA-3L-AA1B-01 | 475 | TCGA-AZ-4684-01 | 1977 | TCGA-D5-6931-01 | 365 |
| TCGA-4N-A93T-01 | 146 | TCGA-AZ-5403-01 | 1910 | TCGA-D5-6932-01 | 346 |
| TCGA-4T-AA8H-01 | 385 | TCGA-AZ-5407-01 | 2683 | TCGA-D5-7000-01 | 312 |
| TCGA-5M-AAT4-01 | 49 | TCGA-AZ-6598-01 | 1503 | TCGA-DM-A0X9-01 | 3641 |
| TCGA-5M-AAT6-01 | 290 | TCGA-AZ-6599-01 | 206 | TCGA-DM-A0XD-01 | 743 |
| TCGA-5M-AATE-01 | 1200 | TCGA-AZ-6601-01 | 3042 | TCGA-DM-A0XF-01 | 1162 |
| TCGA-A6-2675-01 | 1321 | TCGA-AZ-6603-01 | 899 | TCGA-DM-A1D0-01 | 3974 |
| TCGA-A6-2682-01 | 424 | TCGA-AZ-6605-01 | 159 | TCGA-DM-A1D4-01 | 2821 |
| TCGA-A6-2684-01 | 1127 | TCGA-AZ-6606-01 | 357 | TCGA-DM-A1D6-01 | 1518 |
| TCGA-A6-2685-01 | 1133 | TCGA-AZ-6607-01 | 97 | TCGA-DM-A1D7-01 | 405 |
| TCGA-A6-2686-01 | 1126 | TCGA-AZ-6608-01 | 59 | TCGA-DM-A1D8-01 | 383 |
| TCGA-A6-4105-01 | 442 | TCGA-CA-5254-01 | 386 | TCGA-DM-A1D9-01 | 4270 |
| TCGA-A6-5656-01 | 1001 | TCGA-CA-5255-01 | 376 | TCGA-DM-A1DA-01 | 228 |
| TCGA-A6-5657-01 | 962 | TCGA-CA-5256-01 | 379 | TCGA-DM-A1DB-01 | 1348 |
| TCGA-A6-5659-01 | 926 | TCGA-CA-5796-01 | 377 | TCGA-DM-A1HA-01 | 4000 |
| TCGA-A6-5660-01 | 888 | TCGA-CA-5797-01 | 383 | TCGA-DM-A1HB-01 | 4126 |
| TCGA-A6-5661-01 | 1020 | TCGA-CA-6715-01 | 383 | TCGA-DM-A280-01 | 236 |
| TCGA-A6-5662-01 | 718 | TCGA-CA-6716-01 | 371 | TCGA-DM-A282-01 | 4233 |
| TCGA-A6-5664-01 | 672 | TCGA-CA-6717-01 | 388 | TCGA-DM-A285-01 | 179 |
| TCGA-A6-5665-01 | 671 | TCGA-CA-6718-01 | 306 | TCGA-DM-A288-01 | 427 |
| TCGA-A6-5666-01 | 995 | TCGA-CA-6719-01 | 435 | TCGA-DM-A28A-01 | 805 |
| TCGA-A6-5667-01 | 887 | TCGA-CK-4947-01 | 534 | TCGA-DM-A28C-01 | 2475 |
| TCGA-A6-6137-01 | 824 | TCGA-CK-4948-01 | 4502 | TCGA-DM-A28E-01 | 3648 |
| TCGA-A6-6138-01 | 685 | TCGA-CK-4950-01 | 2599 | TCGA-DM-A28F-01 | 1094 |
| TCGA-A6-6140-01 | 734 | TCGA-CK-4951-01 | 2134 | TCGA-DM-A28G-01 | 1849 |
| TCGA-A6-6141-01 | 130 | TCGA-CK-4952-01 | 475 | TCGA-DM-A28H-01 | 3561 |
| TCGA-A6-6142-01 | 763 | TCGA-CK-5912-01 | 1493 | TCGA-DM-A28K-01 | 2988 |
| TCGA-A6-6648-01 | 766 | TCGA-CK-5913-01 | 1561 | TCGA-DM-A28M-01 | 2895 |
| TCGA-A6-6649-01 | 735 | TCGA-CK-5914-01 | 304 | TCGA-F4-6459-01 | 262 |
| TCGA-A6-6650-01 | 627 | TCGA-CK-5916-01 | 643 | TCGA-F4-6460-01 | 972 |
| TCGA-A6-6651-01 | 662 | TCGA-CK-6747-01 | 2523 | TCGA-F4-6461-01 | 338 |
| TCGA-A6-6652-01 | 751 | TCGA-CK-6748-01 | 58 | TCGA-F4-6463-01 | 1087 |
| TCGA-A6-6653-01 | 742 | TCGA-CK-6751-01 | 3780 | TCGA-F4-6569-01 | 1087 |
| TCGA-A6-6654-01 | 726 | TCGA-CM-4743-01 | 701 | TCGA-F4-6570-01 | 188 |
| TCGA-A6-6780-01 | 612 | TCGA-CM-4744-01 | 609 | TCGA-F4-6703-01 | 1456 |
| TCGA-A6-6781-01 | 598 | TCGA-CM-4747-01 | 761 | TCGA-F4-6704-01 | 47 |
| TCGA-A6-6782-01 | 617 | TCGA-CM-4751-01 | 822 | TCGA-F4-6805-01 | 1047 |
| TCGA-A6-A565-01 | 494 | TCGA-CM-5344-01 | 670 | TCGA-F4-6806-01 | 1260 |
| TCGA-A6-A566-01 | 758 | TCGA-CM-5348-01 | 699 | TCGA-F4-6807-01 | 1309 |
| TCGA-A6-A567-01 | 1881 | TCGA-CM-5349-01 | 915 | TCGA-F4-6808-01 | 1024 |
| TCGA-A6-A56B-01 | 1711 | TCGA-CM-5860-01 | 974 | TCGA-F4-6809-01 | 403 |
| TCGA-A6-A5ZU-01 | 293 | TCGA-CM-5861-01 | 457 | TCGA-F4-6854-01 | 16 |
| TCGA-AA-3489-01 | 214 | TCGA-CM-5862-01 | 153 | TCGA-F4-6855-01 | 1442 |
| TCGA-AA-3492-01 | 92 | TCGA-CM-5863-01 | 457 | TCGA-F4-6856-01 | 1074 |
| TCGA-AA-3495-01 | 1127 | TCGA-CM-5864-01 | 457 | TCGA-G4-6293-01 | 4051 |
| TCGA-AA-3496-01 | 31 | TCGA-CM-5868-01 | 518 | TCGA-G4-6294-01 | 858 |
| TCGA-AA-3502-01 | 1065 | TCGA-CM-6161-01 | 457 | TCGA-G4-6295-01 | 254 |
| TCGA-AA-3506-01 | 1765 | TCGA-CM-6162-01 | 365 | TCGA-G4-6297-01 | 2506 |
| TCGA-AA-3509-01 | 1915 | TCGA-CM-6163-01 | 427 | TCGA-G4-6298-01 | 715 |
| TCGA-AA-3511-01 | 212 | TCGA-CM-6164-01 | 883 | TCGA-G4-6299-01 | 2268 |
| TCGA-AA-3526-01 | 580 | TCGA-CM-6165-01 | 488 | TCGA-G4-6302-01 | 2047 |
| TCGA-AA-3655-01 | 1856 | TCGA-CM-6166-01 | 669 | TCGA-G4-6303-01 | 2003 |
| TCGA-AA-3660-01 | 2375 | TCGA-CM-6167-01 | 456 | TCGA-G4-6304-01 | 1631 |
| TCGA-AA-3662-01 | 184 | TCGA-CM-6168-01 | 395 | TCGA-G4-6306-01 | 1359 |
| TCGA-AA-3663-01 | 212 | TCGA-CM-6169-01 | 396 | TCGA-G4-6307-01 | 1674 |
| TCGA-AA-3675-01 | 1431 | TCGA-CM-6170-01 | 457 | TCGA-G4-6309-01 | 2600 |
| TCGA-AA-3685-01 | 1127 | TCGA-CM-6171-01 | 427 | TCGA-G4-6310-01 | 1935 |
| TCGA-AA-3697-01 | 2587 | TCGA-CM-6172-01 | 335 | TCGA-G4-6311-01 | 1199 |
| TCGA-AA-3713-01 | 579 | TCGA-CM-6674-01 | 394 | TCGA-G4-6314-01 | 1093 |
| TCGA-AA-A01P-01 | 1158 | TCGA-CM-6675-01 | 397 | TCGA-G4-6315-01 | 1883 |
| TCGA-AA-A01X-01 | 791 | TCGA-CM-6676-01 | 337 | TCGA-G4-6317-01 | 1095 |
| TCGA-AA-A01Z-01 | 1126 | TCGA-CM-6677-01 | 337 | TCGA-G4-6317-02 | 1095 |
| TCGA-AA-A02K-01 | 426 | TCGA-CM-6678-01 | 335 | TCGA-G4-6320-01 | 804 |
| TCGA-AA-A02Y-01 | 1216 | TCGA-CM-6679-01 | 306 | TCGA-G4-6321-01 | 672 |
| TCGA-AD-5900-01 | 370 | TCGA-CM-6680-01 | 366 | TCGA-G4-6322-01 | 792 |
| TCGA-AD-6548-01 | 650 | TCGA-D5-5537-01 | 1381 | TCGA-G4-6323-01 | 419 |
| TCGA-AD-6888-01 | 472 | TCGA-D5-5538-01 | 1661 | TCGA-G4-6586-01 | 1089 |
| TCGA-AD-6889-01 | 2532 | TCGA-D5-5539-01 | 596 | TCGA-G4-6588-01 | 796 |
| TCGA-AD-6890-01 | 746 | TCGA-D5-5540-01 | 1706 | TCGA-G4-6625-01 | 2792 |
| TCGA-AD-6895-01 | 763 | TCGA-D5-5541-01 | 1701 | TCGA-G4-6626-01 | 1422 |
| TCGA-AD-6899-01 | 176 | TCGA-D5-6529-01 | 614 | TCGA-G4-6627-01 | 2275 |
| TCGA-AD-6901-01 | 682 | TCGA-D5-6530-01 | 621 | TCGA-G4-6628-01 | 2424 |
| TCGA-AD-6963-01 | 834 | TCGA-D5-6531-01 | 540 | TCGA-NH-A50T-01 | 553 |
| TCGA-AD-6964-01 | 331 | TCGA-D5-6532-01 | 555 | TCGA-NH-A50U-01 | 334 |
| TCGA-AD-6965-01 | 805 | TCGA-D5-6533-01 | 775 | TCGA-NH-A50V-01 | 588 |
| TCGA-AD-A5EK-01 | 500 | TCGA-D5-6534-01 | 1316 | TCGA-NH-A5IV-01 | 588 |
| TCGA-AM-5820-01 | 14 | TCGA-D5-6535-01 | 460 | TCGA-NH-A6GA-01 | 302 |
| TCGA-AM-5821-01 | 28 | TCGA-D5-6536-01 | 543 | TCGA-NH-A6GB-01 | 476 |
| TCGA-AU-6004-01 | 824 | TCGA-D5-6537-01 | 146 | TCGA-NH-A6GC-01 | 389 |
| TCGA-AY-5543-01 | 1004 | TCGA-D5-6538-01 | 521 | TCGA-NH-A8F7-01 | 543 |
| TCGA-AY-6197-01 | 652 | TCGA-D5-6539-01 | 380 | TCGA-NH-A8F7-06 | 543 |
| TCGA-AY-6386-01 | 542 | TCGA-D5-6540-01 | 491 | TCGA-NH-A8F8-01 | 511 |
| TCGA-AY-A54L-01 | 525 | TCGA-D5-6541-01 | 474 | TCGA-QG-A5YV-01 | 1301 |
| TCGA-AY-A69D-01 | 543 | TCGA-D5-6898-01 | 229 | TCGA-QG-A5YW-01 | 896 |
| TCGA-AY-A71X-01 | 588 | TCGA-D5-6920-01 | 377 | TCGA-QG-A5YX-01 | 1003 |
| TCGA-AY-A8YK-01 | 573 | TCGA-D5-6922-01 | 308 | TCGA-QG-A5Z1-01 | 256 |
| TCGA-AZ-4313-01 | 2310 | TCGA-D5-6923-01 | 378 | TCGA-QG-A5Z2-01 | 952 |
| TCGA-AZ-4315-01 | 1776 | TCGA-D5-6924-01 | 435 | TCGA-QL-A97D-01 | 666 |
| TCGA-AZ-4323-01 | 43 | TCGA-D5-6926-01 | 275 | TCGA-RU-A8FL-01 | 1177 |
| TCGA-AZ-4614-01 | 172 | TCGA-D5-6928-01 | 354 | TCGA-SS-A7HO-01 | 1829 |
| TCGA-AZ-4615-01 | 1002 | TCGA-D5-6929-01 | 408 | TCGA-T9-A92H-01 | 362 |
| TCGA-AZ-4616-01 | 156 | TCGA-D5-6930-01 | 406 | TCGA-WS-AB45-01 | 2130 |
| TCGA-AZ-4682-01 | 680 |  |  |  |  |

^a^ Column 1 to 2 represents samples and OS respectively.

**Supplementary Table 1b. TCGA patients samples for exon expression ^a^.**

| ***sample*** | ***OS*** | ***sample*** | ***OS*** | ***sample*** | ***OS*** |
| --- | --- | --- | --- | --- | --- |
| TCGA-3L-AA1B-01 | 475 | TCGA-AZ-4684-01 | 1977 | TCGA-D5-6931-01 | 365 |
| TCGA-4N-A93T-01 | 146 | TCGA-AZ-5403-01 | 1910 | TCGA-D5-6932-01 | 346 |
| TCGA-4T-AA8H-01 | 385 | TCGA-AZ-5407-01 | 2683 | TCGA-D5-7000-01 | 312 |
| TCGA-5M-AAT4-01 | 49 | TCGA-AZ-6598-01 | 1503 | TCGA-DM-A0X9-01 | 3641 |
| TCGA-5M-AAT6-01 | 290 | TCGA-AZ-6599-01 | 206 | TCGA-DM-A0XD-01 | 743 |
| TCGA-5M-AATE-01 | 1200 | TCGA-AZ-6601-01 | 3042 | TCGA-DM-A0XF-01 | 1162 |
| TCGA-A6-2675-01 | 1321 | TCGA-AZ-6603-01 | 899 | TCGA-DM-A1D0-01 | 3974 |
| TCGA-A6-2682-01 | 424 | TCGA-AZ-6605-01 | 159 | TCGA-DM-A1D4-01 | 2821 |
| TCGA-A6-2684-01 | 1127 | TCGA-AZ-6606-01 | 357 | TCGA-DM-A1D6-01 | 1518 |
| TCGA-A6-2685-01 | 1133 | TCGA-AZ-6607-01 | 97 | TCGA-DM-A1D7-01 | 405 |
| TCGA-A6-2686-01 | 1126 | TCGA-AZ-6608-01 | 59 | TCGA-DM-A1D8-01 | 383 |
| TCGA-A6-4105-01 | 442 | TCGA-CA-5254-01 | 386 | TCGA-DM-A1D9-01 | 4270 |
| TCGA-A6-5656-01 | 1001 | TCGA-CA-5255-01 | 376 | TCGA-DM-A1DA-01 | 228 |
| TCGA-A6-5657-01 | 962 | TCGA-CA-5256-01 | 379 | TCGA-DM-A1DB-01 | 1348 |
| TCGA-A6-5659-01 | 926 | TCGA-CA-5796-01 | 377 | TCGA-DM-A1HA-01 | 4000 |
| TCGA-A6-5660-01 | 888 | TCGA-CA-5797-01 | 383 | TCGA-DM-A1HB-01 | 4126 |
| TCGA-A6-5661-01 | 1020 | TCGA-CA-6715-01 | 383 | TCGA-DM-A280-01 | 236 |
| TCGA-A6-5662-01 | 718 | TCGA-CA-6716-01 | 371 | TCGA-DM-A282-01 | 4233 |
| TCGA-A6-5664-01 | 672 | TCGA-CA-6717-01 | 388 | TCGA-DM-A285-01 | 179 |
| TCGA-A6-5665-01 | 671 | TCGA-CA-6718-01 | 306 | TCGA-DM-A288-01 | 427 |
| TCGA-A6-5666-01 | 995 | TCGA-CA-6719-01 | 435 | TCGA-DM-A28A-01 | 805 |
| TCGA-A6-5667-01 | 887 | TCGA-CK-4947-01 | 534 | TCGA-DM-A28C-01 | 2475 |
| TCGA-A6-6137-01 | 824 | TCGA-CK-4948-01 | 4502 | TCGA-DM-A28E-01 | 3648 |
| TCGA-A6-6138-01 | 685 | TCGA-CK-4950-01 | 2599 | TCGA-DM-A28F-01 | 1094 |
| TCGA-A6-6140-01 | 734 | TCGA-CK-4951-01 | 2134 | TCGA-DM-A28G-01 | 1849 |
| TCGA-A6-6141-01 | 130 | TCGA-CK-4952-01 | 475 | TCGA-DM-A28H-01 | 3561 |
| TCGA-A6-6142-01 | 763 | TCGA-CK-5912-01 | 1493 | TCGA-DM-A28K-01 | 2988 |
| TCGA-A6-6648-01 | 766 | TCGA-CK-5913-01 | 1561 | TCGA-DM-A28M-01 | 2895 |
| TCGA-A6-6649-01 | 735 | TCGA-CK-5914-01 | 304 | TCGA-F4-6459-01 | 262 |
| TCGA-A6-6650-01 | 627 | TCGA-CK-5916-01 | 643 | TCGA-F4-6460-01 | 972 |
| TCGA-A6-6651-01 | 662 | TCGA-CK-6747-01 | 2523 | TCGA-F4-6461-01 | 338 |
| TCGA-A6-6652-01 | 751 | TCGA-CK-6748-01 | 58 | TCGA-F4-6463-01 | 1087 |
| TCGA-A6-6653-01 | 742 | TCGA-CK-6751-01 | 3780 | TCGA-F4-6569-01 | 1087 |
| TCGA-A6-6654-01 | 726 | TCGA-CM-4743-01 | 701 | TCGA-F4-6570-01 | 188 |
| TCGA-A6-6780-01 | 612 | TCGA-CM-4744-01 | 609 | TCGA-F4-6703-01 | 1456 |
| TCGA-A6-6781-01 | 598 | TCGA-CM-4747-01 | 761 | TCGA-F4-6704-01 | 47 |
| TCGA-A6-6782-01 | 617 | TCGA-CM-4751-01 | 822 | TCGA-F4-6805-01 | 1047 |
| TCGA-A6-A565-01 | 494 | TCGA-CM-5344-01 | 670 | TCGA-F4-6806-01 | 1260 |
| TCGA-A6-A566-01 | 758 | TCGA-CM-5348-01 | 699 | TCGA-F4-6807-01 | 1309 |
| TCGA-A6-A567-01 | 1881 | TCGA-CM-5349-01 | 915 | TCGA-F4-6808-01 | 1024 |
| TCGA-A6-A56B-01 | 1711 | TCGA-CM-5860-01 | 974 | TCGA-F4-6809-01 | 403 |
| TCGA-A6-A5ZU-01 | 293 | TCGA-CM-5861-01 | 457 | TCGA-F4-6854-01 | 16 |
| TCGA-AA-3489-01 | 214 | TCGA-CM-5862-01 | 153 | TCGA-F4-6855-01 | 1442 |
| TCGA-AA-3492-01 | 92 | TCGA-CM-5863-01 | 457 | TCGA-F4-6856-01 | 1074 |
| TCGA-AA-3495-01 | 1127 | TCGA-CM-5864-01 | 457 | TCGA-G4-6293-01 | 4051 |
| TCGA-AA-3496-01 | 31 | TCGA-CM-5868-01 | 518 | TCGA-G4-6294-01 | 858 |
| TCGA-AA-3502-01 | 1065 | TCGA-CM-6161-01 | 457 | TCGA-G4-6295-01 | 254 |
| TCGA-AA-3506-01 | 1765 | TCGA-CM-6162-01 | 365 | TCGA-G4-6297-01 | 2506 |
| TCGA-AA-3509-01 | 1915 | TCGA-CM-6163-01 | 427 | TCGA-G4-6298-01 | 715 |
| TCGA-AA-3511-01 | 212 | TCGA-CM-6164-01 | 883 | TCGA-G4-6299-01 | 2268 |
| TCGA-AA-3526-01 | 580 | TCGA-CM-6165-01 | 488 | TCGA-G4-6302-01 | 2047 |
| TCGA-AA-3655-01 | 1856 | TCGA-CM-6166-01 | 669 | TCGA-G4-6303-01 | 2003 |
| TCGA-AA-3660-01 | 2375 | TCGA-CM-6167-01 | 456 | TCGA-G4-6304-01 | 1631 |
| TCGA-AA-3662-01 | 184 | TCGA-CM-6168-01 | 395 | TCGA-G4-6306-01 | 1359 |
| TCGA-AA-3663-01 | 212 | TCGA-CM-6169-01 | 396 | TCGA-G4-6307-01 | 1674 |
| TCGA-AA-3675-01 | 1431 | TCGA-CM-6170-01 | 457 | TCGA-G4-6309-01 | 2600 |
| TCGA-AA-3685-01 | 1127 | TCGA-CM-6171-01 | 427 | TCGA-G4-6310-01 | 1935 |
| TCGA-AA-3697-01 | 2587 | TCGA-CM-6172-01 | 335 | TCGA-G4-6311-01 | 1199 |
| TCGA-AA-3713-01 | 579 | TCGA-CM-6674-01 | 394 | TCGA-G4-6314-01 | 1093 |
| TCGA-AA-A01P-01 | 1158 | TCGA-CM-6675-01 | 397 | TCGA-G4-6315-01 | 1883 |
| TCGA-AA-A01X-01 | 791 | TCGA-CM-6676-01 | 337 | TCGA-G4-6317-01 | 1095 |
| TCGA-AA-A01Z-01 | 1126 | TCGA-CM-6677-01 | 337 | TCGA-G4-6317-02 | 1095 |
| TCGA-AA-A02K-01 | 426 | TCGA-CM-6678-01 | 335 | TCGA-G4-6320-01 | 804 |
| TCGA-AA-A02Y-01 | 1216 | TCGA-CM-6679-01 | 306 | TCGA-G4-6321-01 | 672 |
| TCGA-AD-5900-01 | 370 | TCGA-CM-6680-01 | 366 | TCGA-G4-6322-01 | 792 |
| TCGA-AD-6548-01 | 650 | TCGA-D5-5537-01 | 1381 | TCGA-G4-6323-01 | 419 |
| TCGA-AD-6888-01 | 472 | TCGA-D5-5538-01 | 1661 | TCGA-G4-6586-01 | 1089 |
| TCGA-AD-6889-01 | 2532 | TCGA-D5-5539-01 | 596 | TCGA-G4-6588-01 | 796 |
| TCGA-AD-6890-01 | 746 | TCGA-D5-5540-01 | 1706 | TCGA-G4-6625-01 | 2792 |
| TCGA-AD-6895-01 | 763 | TCGA-D5-5541-01 | 1701 | TCGA-G4-6626-01 | 1422 |
| TCGA-AD-6899-01 | 176 | TCGA-D5-6529-01 | 614 | TCGA-G4-6627-01 | 2275 |
| TCGA-AD-6901-01 | 682 | TCGA-D5-6530-01 | 621 | TCGA-G4-6628-01 | 2424 |
| TCGA-AD-6963-01 | 834 | TCGA-D5-6531-01 | 540 | TCGA-NH-A50T-01 | 553 |
| TCGA-AD-6964-01 | 331 | TCGA-D5-6532-01 | 555 | TCGA-NH-A50U-01 | 334 |
| TCGA-AD-6965-01 | 805 | TCGA-D5-6533-01 | 775 | TCGA-NH-A50V-01 | 588 |
| TCGA-AD-A5EK-01 | 500 | TCGA-D5-6534-01 | 1316 | TCGA-NH-A5IV-01 | 588 |
| TCGA-AM-5820-01 | 14 | TCGA-D5-6535-01 | 460 | TCGA-NH-A6GA-01 | 302 |
| TCGA-AM-5821-01 | 28 | TCGA-D5-6536-01 | 543 | TCGA-NH-A6GB-01 | 476 |
| TCGA-AU-6004-01 | 824 | TCGA-D5-6537-01 | 146 | TCGA-NH-A6GC-01 | 389 |
| TCGA-AY-5543-01 | 1004 | TCGA-D5-6538-01 | 521 | TCGA-NH-A8F7-01 | 543 |
| TCGA-AY-6197-01 | 652 | TCGA-D5-6539-01 | 380 | TCGA-NH-A8F7-06 | 543 |
| TCGA-AY-6386-01 | 542 | TCGA-D5-6540-01 | 491 | TCGA-NH-A8F8-01 | 511 |
| TCGA-AY-A54L-01 | 525 | TCGA-D5-6541-01 | 474 | TCGA-QG-A5YV-01 | 1301 |
| TCGA-AY-A69D-01 | 543 | TCGA-D5-6898-01 | 229 | TCGA-QG-A5YW-01 | 896 |
| TCGA-AY-A71X-01 | 588 | TCGA-D5-6920-01 | 377 | TCGA-QG-A5YX-01 | 1003 |
| TCGA-AY-A8YK-01 | 573 | TCGA-D5-6922-01 | 308 | TCGA-QG-A5Z1-01 | 256 |
| TCGA-AZ-4313-01 | 2310 | TCGA-D5-6923-01 | 378 | TCGA-QG-A5Z2-01 | 952 |
| TCGA-AZ-4315-01 | 1776 | TCGA-D5-6924-01 | 435 | TCGA-QL-A97D-01 | 666 |
| TCGA-AZ-4323-01 | 43 | TCGA-D5-6926-01 | 275 | TCGA-RU-A8FL-01 | 1177 |
| TCGA-AZ-4614-01 | 172 | TCGA-D5-6928-01 | 354 | TCGA-SS-A7HO-01 | 1829 |
| TCGA-AZ-4615-01 | 1002 | TCGA-D5-6929-01 | 408 | TCGA-T9-A92H-01 | 362 |
| TCGA-AZ-4616-01 | 156 | TCGA-D5-6930-01 | 406 | TCGA-WS-AB45-01 | 2130 |
| TCGA-AZ-4682-01 | 680 |  |  |  |  |

^a^ Column 1 to 2 represents samples and OS respectively.

**Supplementary Table 1c. TCGA patients samples for DNA methylation ^a^.**

| ***sample*** | ***OS*** | ***sample*** | ***OS*** | ***sample*** | ***OS*** |
| --- | --- | --- | --- | --- | --- |
| TCGA.3L.AA1B.01 | 475 | TCGA.AZ.4616.01 | 156 | TCGA.D5.6926.01 | 275 |
| TCGA.4N.A93T.01 | 146 | TCGA.AZ.4681.01 | 3247 | TCGA.D5.6928.01 | 354 |
| TCGA.4T.AA8H.01 | 385 | TCGA.AZ.4682.01 | 680 | TCGA.D5.6929.01 | 408 |
| TCGA.5M.AAT4.01 | 49 | TCGA.AZ.4684.01 | 1977 | TCGA.D5.6930.01 | 406 |
| TCGA.5M.AAT6.01 | 290 | TCGA.AZ.5403.01 | 1910 | TCGA.D5.6931.01 | 365 |
| TCGA.5M.AATE.01 | 1200 | TCGA.AZ.5407.01 | 2683 | TCGA.D5.6932.01 | 346 |
| TCGA.A6.2671.01 | 1331 | TCGA.AZ.6598.01 | 1503 | TCGA.D5.7000.01 | 312 |
| TCGA.A6.2672.01 | 1419 | TCGA.AZ.6599.01 | 206 | TCGA.DM.A0X9.01 | 3641 |
| TCGA.A6.2675.01 | 1321 | TCGA.AZ.6601.01 | 3042 | TCGA.DM.A0XD.01 | 743 |
| TCGA.A6.2677.01 | 740 | TCGA.AZ.6603.01 | 899 | TCGA.DM.A0XF.01 | 1162 |
| TCGA.A6.2679.01 | 1366 | TCGA.AZ.6605.01 | 159 | TCGA.DM.A1D0.01 | 3974 |
| TCGA.A6.2680.01 | 1068 | TCGA.AZ.6606.01 | 357 | TCGA.DM.A1D4.01 | 2821 |
| TCGA.A6.2681.01 | 1387 | TCGA.AZ.6607.01 | 97 | TCGA.DM.A1D6.01 | 1518 |
| TCGA.A6.2682.01 | 424 | TCGA.AZ.6608.01 | 59 | TCGA.DM.A1D7.01 | 405 |
| TCGA.A6.2684.01 | 1127 | TCGA.CA.5254.01 | 386 | TCGA.DM.A1D8.01 | 383 |
| TCGA.A6.2685.01 | 1133 | TCGA.CA.5255.01 | 376 | TCGA.DM.A1D9.01 | 4270 |
| TCGA.A6.2686.01 | 1126 | TCGA.CA.5256.01 | 379 | TCGA.DM.A1DA.01 | 228 |
| TCGA.A6.3809.01 | 996 | TCGA.CA.5796.01 | 377 | TCGA.DM.A1DB.01 | 1348 |
| TCGA.A6.3810.01 | 1111 | TCGA.CA.5797.01 | 383 | TCGA.DM.A1HA.01 | 4000 |
| TCGA.A6.4105.01 | 442 | TCGA.CA.6715.01 | 383 | TCGA.DM.A1HB.01 | 4126 |
| TCGA.A6.4107.01 | 987 | TCGA.CA.6716.01 | 371 | TCGA.DM.A280.01 | 236 |
| TCGA.A6.5656.01 | 1001 | TCGA.CA.6717.01 | 388 | TCGA.DM.A282.01 | 4233 |
| TCGA.A6.5657.01 | 962 | TCGA.CA.6718.01 | 306 | TCGA.DM.A285.01 | 179 |
| TCGA.A6.5659.01 | 926 | TCGA.CA.6719.01 | 435 | TCGA.DM.A288.01 | 427 |
| TCGA.A6.5660.01 | 888 | TCGA.CK.4947.01 | 534 | TCGA.DM.A28A.01 | 805 |
| TCGA.A6.5661.01 | 1020 | TCGA.CK.4948.01 | 4502 | TCGA.DM.A28C.01 | 2475 |
| TCGA.A6.5662.01 | 718 | TCGA.CK.4950.01 | 2599 | TCGA.DM.A28E.01 | 3648 |
| TCGA.A6.5664.01 | 672 | TCGA.CK.4951.01 | 2134 | TCGA.DM.A28F.01 | 1094 |
| TCGA.A6.5665.01 | 671 | TCGA.CK.4952.01 | 475 | TCGA.DM.A28G.01 | 1849 |
| TCGA.A6.5666.01 | 995 | TCGA.CK.5912.01 | 1493 | TCGA.DM.A28H.01 | 3561 |
| TCGA.A6.5667.01 | 887 | TCGA.CK.5913.01 | 1561 | TCGA.DM.A28K.01 | 2988 |
| TCGA.A6.6137.01 | 824 | TCGA.CK.5914.01 | 304 | TCGA.DM.A28M.01 | 2895 |
| TCGA.A6.6138.01 | 685 | TCGA.CK.5916.01 | 643 | TCGA.F4.6459.01 | 262 |
| TCGA.A6.6140.01 | 734 | TCGA.CK.6747.01 | 2523 | TCGA.F4.6460.01 | 972 |
| TCGA.A6.6141.01 | 130 | TCGA.CK.6748.01 | 58 | TCGA.F4.6461.01 | 338 |
| TCGA.A6.6142.01 | 763 | TCGA.CK.6751.01 | 3780 | TCGA.F4.6463.01 | 1087 |
| TCGA.A6.6648.01 | 766 | TCGA.CM.4743.01 | 701 | TCGA.F4.6569.01 | 1087 |
| TCGA.A6.6649.01 | 735 | TCGA.CM.4744.01 | 609 | TCGA.F4.6570.01 | 188 |
| TCGA.A6.6650.01 | 627 | TCGA.CM.4746.01 | 1126 | TCGA.F4.6703.01 | 1456 |
| TCGA.A6.6651.01 | 662 | TCGA.CM.4747.01 | 761 | TCGA.F4.6704.01 | 47 |
| TCGA.A6.6652.01 | 751 | TCGA.CM.4748.01 | 792 | TCGA.F4.6805.01 | 1047 |
| TCGA.A6.6653.01 | 742 | TCGA.CM.4750.01 | 244 | TCGA.F4.6806.01 | 1260 |
| TCGA.A6.6654.01 | 726 | TCGA.CM.4751.01 | 822 | TCGA.F4.6807.01 | 1309 |
| TCGA.A6.6780.01 | 612 | TCGA.CM.4752.01 | 396 | TCGA.F4.6808.01 | 1024 |
| TCGA.A6.6781.01 | 598 | TCGA.CM.5341.01 | 884 | TCGA.F4.6809.01 | 403 |
| TCGA.A6.6782.01 | 617 | TCGA.CM.5344.01 | 670 | TCGA.F4.6854.01 | 16 |
| TCGA.A6.A565.01 | 494 | TCGA.CM.5348.01 | 699 | TCGA.F4.6855.01 | 1442 |
| TCGA.A6.A566.01 | 758 | TCGA.CM.5349.01 | 915 | TCGA.F4.6856.01 | 1074 |
| TCGA.A6.A567.01 | 1881 | TCGA.CM.5860.01 | 974 | TCGA.G4.6293.01 | 4051 |
| TCGA.A6.A56B.01 | 1711 | TCGA.CM.5861.01 | 457 | TCGA.G4.6294.01 | 858 |
| TCGA.A6.A5ZU.01 | 293 | TCGA.CM.5862.01 | 153 | TCGA.G4.6295.01 | 254 |
| TCGA.AA.3488.01 | 153 | TCGA.CM.5863.01 | 457 | TCGA.G4.6297.01 | 2506 |
| TCGA.AA.3489.01 | 214 | TCGA.CM.5864.01 | 457 | TCGA.G4.6298.01 | 715 |
| TCGA.AA.3492.01 | 92 | TCGA.CM.5868.01 | 518 | TCGA.G4.6299.01 | 2268 |
| TCGA.AA.3494.01 | 31 | TCGA.CM.6161.01 | 457 | TCGA.G4.6302.01 | 2047 |
| TCGA.AA.3495.01 | 1127 | TCGA.CM.6162.01 | 365 | TCGA.G4.6303.01 | 2003 |
| TCGA.AA.3496.01 | 31 | TCGA.CM.6163.01 | 427 | TCGA.G4.6304.01 | 1631 |
| TCGA.AA.3502.01 | 1065 | TCGA.CM.6164.01 | 883 | TCGA.G4.6306.01 | 1359 |
| TCGA.AA.3506.01 | 1765 | TCGA.CM.6165.01 | 488 | TCGA.G4.6307.01 | 1674 |
| TCGA.AA.3509.01 | 1915 | TCGA.CM.6166.01 | 669 | TCGA.G4.6309.01 | 2600 |
| TCGA.AA.3510.01 | 1946 | TCGA.CM.6167.01 | 456 | TCGA.G4.6310.01 | 1935 |
| TCGA.AA.3511.01 | 212 | TCGA.CM.6168.01 | 395 | TCGA.G4.6311.01 | 1199 |
| TCGA.AA.3655.01 | 1856 | TCGA.CM.6169.01 | 396 | TCGA.G4.6314.01 | 1093 |
| TCGA.AA.3660.01 | 2375 | TCGA.CM.6170.01 | 457 | TCGA.G4.6315.01 | 1883 |
| TCGA.AA.3662.01 | 184 | TCGA.CM.6171.01 | 427 | TCGA.G4.6317.01 | 1095 |
| TCGA.AA.3663.01 | 212 | TCGA.CM.6172.01 | 335 | TCGA.G4.6317.02 | 1095 |
| TCGA.AA.3697.01 | 2587 | TCGA.CM.6674.01 | 394 | TCGA.G4.6320.01 | 804 |
| TCGA.AA.3713.01 | 579 | TCGA.CM.6675.01 | 397 | TCGA.G4.6321.01 | 672 |
| TCGA.AD.5900.01 | 370 | TCGA.CM.6676.01 | 337 | TCGA.G4.6322.01 | 792 |
| TCGA.AD.6548.01 | 650 | TCGA.CM.6677.01 | 337 | TCGA.G4.6323.01 | 419 |
| TCGA.AD.6888.01 | 472 | TCGA.CM.6678.01 | 335 | TCGA.G4.6586.01 | 1089 |
| TCGA.AD.6889.01 | 2532 | TCGA.CM.6679.01 | 306 | TCGA.G4.6588.01 | 796 |
| TCGA.AD.6890.01 | 746 | TCGA.CM.6680.01 | 366 | TCGA.G4.6625.01 | 2792 |
| TCGA.AD.6895.01 | 763 | TCGA.D5.5537.01 | 1381 | TCGA.G4.6626.01 | 1422 |
| TCGA.AD.6899.01 | 176 | TCGA.D5.5538.01 | 1661 | TCGA.G4.6627.01 | 2275 |
| TCGA.AD.6901.01 | 682 | TCGA.D5.5539.01 | 596 | TCGA.G4.6628.01 | 2424 |
| TCGA.AD.6963.01 | 834 | TCGA.D5.5540.01 | 1706 | TCGA.NH.A50T.01 | 553 |
| TCGA.AD.6964.01 | 331 | TCGA.D5.5541.01 | 1701 | TCGA.NH.A50U.01 | 334 |
| TCGA.AD.6965.01 | 805 | TCGA.D5.6529.01 | 614 | TCGA.NH.A50V.01 | 588 |
| TCGA.AD.A5EK.01 | 500 | TCGA.D5.6530.01 | 621 | TCGA.NH.A5IV.01 | 588 |
| TCGA.AM.5820.01 | 14 | TCGA.D5.6531.01 | 540 | TCGA.NH.A6GA.01 | 302 |
| TCGA.AM.5821.01 | 28 | TCGA.D5.6532.01 | 555 | TCGA.NH.A6GB.01 | 476 |
| TCGA.AU.6004.01 | 824 | TCGA.D5.6533.01 | 775 | TCGA.NH.A6GC.01 | 389 |
| TCGA.AY.5543.01 | 1004 | TCGA.D5.6534.01 | 1316 | TCGA.NH.A8F7.01 | 543 |
| TCGA.AY.6197.01 | 652 | TCGA.D5.6535.01 | 460 | TCGA.NH.A8F7.06 | 543 |
| TCGA.AY.6386.01 | 542 | TCGA.D5.6536.01 | 543 | TCGA.NH.A8F8.01 | 511 |
| TCGA.AY.A54L.01 | 525 | TCGA.D5.6537.01 | 146 | TCGA.QG.A5YV.01 | 1301 |
| TCGA.AY.A69D.01 | 543 | TCGA.D5.6538.01 | 521 | TCGA.QG.A5YW.01 | 896 |
| TCGA.AY.A71X.01 | 588 | TCGA.D5.6539.01 | 380 | TCGA.QG.A5YX.01 | 1003 |
| TCGA.AY.A8YK.01 | 573 | TCGA.D5.6540.01 | 491 | TCGA.QG.A5Z1.01 | 256 |
| TCGA.AZ.4308.01 | 3324 | TCGA.D5.6541.01 | 474 | TCGA.QG.A5Z2.01 | 952 |
| TCGA.AZ.4313.01 | 2310 | TCGA.D5.6898.01 | 229 | TCGA.QL.A97D.01 | 666 |
| TCGA.AZ.4315.01 | 1776 | TCGA.D5.6920.01 | 377 | TCGA.RU.A8FL.01 | 1177 |
| TCGA.AZ.4323.01 | 43 | TCGA.D5.6922.01 | 308 | TCGA.SS.A7HO.01 | 1829 |
| TCGA.AZ.4614.01 | 172 | TCGA.D5.6923.01 | 378 | TCGA.T9.A92H.01 | 362 |
| TCGA.AZ.4615.01 | 1002 | TCGA.D5.6924.01 | 435 | TCGA.WS.AB45.01 | 2130 |

^a^ Column 1 to 2 represents samples and OS respectively.

**Supplementary Table 1d. TCGA patients samples for somatic mutations ^a^.**

| ***sample*** | ***OS*** | ***sample*** | ***OS*** | ***sample*** | ***OS*** |
| --- | --- | --- | --- | --- | --- |
| TCGA-A6-2671-01 | 1331 | TCGA-CA-5796-01 | 377 | TCGA-D5-6926-01 | 275 |
| TCGA-A6-2675-01 | 1321 | TCGA-CA-5797-01 | 383 | TCGA-D5-6928-01 | 354 |
| TCGA-A6-4105-01 | 442 | TCGA-CA-6716-01 | 371 | TCGA-D5-6929-01 | 408 |
| TCGA-A6-5656-01 | 1001 | TCGA-CA-6717-01 | 388 | TCGA-D5-6930-01 | 406 |
| TCGA-A6-5657-01 | 962 | TCGA-CA-6718-01 | 306 | TCGA-D5-6931-01 | 365 |
| TCGA-A6-5659-01 | 926 | TCGA-CA-6719-01 | 435 | TCGA-D5-6932-01 | 346 |
| TCGA-A6-5660-01 | 888 | TCGA-CK-4947-01 | 534 | TCGA-D5-7000-01 | 312 |
| TCGA-A6-5661-01 | 1020 | TCGA-CK-4948-01 | 4502 | TCGA-DM-A0X9-01 | 3641 |
| TCGA-A6-5662-01 | 718 | TCGA-CK-4950-01 | 2599 | TCGA-DM-A0XD-01 | 743 |
| TCGA-A6-5664-01 | 672 | TCGA-CK-4952-01 | 475 | TCGA-DM-A0XF-01 | 1162 |
| TCGA-A6-5665-01 | 671 | TCGA-CK-5912-01 | 1493 | TCGA-DM-A1D0-01 | 3974 |
| TCGA-A6-5666-01 | 995 | TCGA-CK-5913-01 | 1561 | TCGA-DM-A1D4-01 | 2821 |
| TCGA-A6-5667-01 | 887 | TCGA-CK-5914-01 | 304 | TCGA-DM-A1D6-01 | 1518 |
| TCGA-A6-6137-01 | 824 | TCGA-CK-5916-01 | 643 | TCGA-DM-A1D7-01 | 405 |
| TCGA-A6-6138-01 | 685 | TCGA-CM-4743-01 | 701 | TCGA-DM-A1D8-01 | 383 |
| TCGA-A6-6140-01 | 734 | TCGA-CM-4744-01 | 609 | TCGA-DM-A1D9-01 | 4270 |
| TCGA-A6-6141-01 | 130 | TCGA-CM-4746-01 | 1126 | TCGA-DM-A1DA-01 | 228 |
| TCGA-A6-6142-01 | 763 | TCGA-CM-4747-01 | 761 | TCGA-DM-A1DB-01 | 1348 |
| TCGA-A6-6648-01 | 766 | TCGA-CM-4748-01 | 792 | TCGA-DM-A1HA-01 | 4000 |
| TCGA-A6-6649-01 | 735 | TCGA-CM-4750-01 | 244 | TCGA-DM-A282-01 | 4233 |
| TCGA-A6-6650-01 | 627 | TCGA-CM-4752-01 | 396 | TCGA-DM-A285-01 | 179 |
| TCGA-A6-6651-01 | 662 | TCGA-CM-5341-01 | 884 | TCGA-DM-A28C-01 | 2475 |
| TCGA-A6-6652-01 | 751 | TCGA-CM-5344-01 | 670 | TCGA-DM-A28E-01 | 3648 |
| TCGA-A6-6653-01 | 742 | TCGA-CM-5348-01 | 699 | TCGA-DM-A28F-01 | 1094 |
| TCGA-A6-6654-01 | 726 | TCGA-CM-5349-01 | 915 | TCGA-DM-A28G-01 | 1849 |
| TCGA-A6-6780-01 | 612 | TCGA-CM-5860-01 | 974 | TCGA-DM-A28H-01 | 3561 |
| TCGA-A6-6781-01 | 598 | TCGA-CM-5861-01 | 457 | TCGA-DM-A28K-01 | 2988 |
| TCGA-A6-6782-01 | 617 | TCGA-CM-5862-01 | 153 | TCGA-DM-A28M-01 | 2895 |
| TCGA-AA-3489-01 | 214 | TCGA-CM-5863-01 | 457 | TCGA-F4-6459-01 | 262 |
| TCGA-AA-3492-01 | 92 | TCGA-CM-5864-01 | 457 | TCGA-F4-6460-01 | 972 |
| TCGA-AA-3502-01 | 1065 | TCGA-CM-5868-01 | 518 | TCGA-F4-6461-01 | 338 |
| TCGA-AA-3510-01 | 1946 | TCGA-CM-6161-01 | 457 | TCGA-F4-6463-01 | 1087 |
| TCGA-AA-3511-01 | 212 | TCGA-CM-6162-01 | 365 | TCGA-F4-6569-01 | 1087 |
| TCGA-AA-3655-01 | 1856 | TCGA-CM-6163-01 | 427 | TCGA-F4-6570-01 | 188 |
| TCGA-AA-3660-01 | 2375 | TCGA-CM-6164-01 | 883 | TCGA-F4-6703-01 | 1456 |
| TCGA-AA-3662-01 | 184 | TCGA-CM-6165-01 | 488 | TCGA-F4-6704-01 | 47 |
| TCGA-AA-3663-01 | 212 | TCGA-CM-6166-01 | 669 | TCGA-F4-6805-01 | 1047 |
| TCGA-AA-3697-01 | 2587 | TCGA-CM-6168-01 | 395 | TCGA-F4-6806-01 | 1260 |
| TCGA-AA-3713-01 | 579 | TCGA-CM-6169-01 | 396 | TCGA-F4-6807-01 | 1309 |
| TCGA-AD-5900-01 | 370 | TCGA-CM-6170-01 | 457 | TCGA-F4-6808-01 | 1024 |
| TCGA-AD-6548-01 | 650 | TCGA-CM-6171-01 | 427 | TCGA-F4-6809-01 | 403 |
| TCGA-AD-6888-01 | 472 | TCGA-CM-6172-01 | 335 | TCGA-F4-6854-01 | 16 |
| TCGA-AD-6889-01 | 2532 | TCGA-CM-6674-01 | 394 | TCGA-F4-6855-01 | 1442 |
| TCGA-AD-6890-01 | 746 | TCGA-CM-6675-01 | 397 | TCGA-F4-6856-01 | 1074 |
| TCGA-AD-6895-01 | 763 | TCGA-CM-6676-01 | 337 | TCGA-G4-6293-01 | 4051 |
| TCGA-AD-6899-01 | 176 | TCGA-CM-6677-01 | 337 | TCGA-G4-6294-01 | 858 |
| TCGA-AD-6901-01 | 682 | TCGA-CM-6678-01 | 335 | TCGA-G4-6295-01 | 254 |
| TCGA-AD-6963-01 | 834 | TCGA-CM-6679-01 | 306 | TCGA-G4-6297-01 | 2506 |
| TCGA-AD-6964-01 | 331 | TCGA-CM-6680-01 | 366 | TCGA-G4-6298-01 | 715 |
| TCGA-AD-6965-01 | 805 | TCGA-D5-5537-01 | 1381 | TCGA-G4-6299-01 | 2268 |
| TCGA-AM-5820-01 | 14 | TCGA-D5-5538-01 | 1661 | TCGA-G4-6302-01 | 2047 |
| TCGA-AM-5821-01 | 28 | TCGA-D5-5539-01 | 596 | TCGA-G4-6303-01 | 2003 |
| TCGA-AU-6004-01 | 824 | TCGA-D5-5540-01 | 1706 | TCGA-G4-6304-01 | 1631 |
| TCGA-AY-5543-01 | 1004 | TCGA-D5-5541-01 | 1701 | TCGA-G4-6306-01 | 1359 |
| TCGA-AY-6197-01 | 652 | TCGA-D5-6529-01 | 614 | TCGA-G4-6307-01 | 1674 |
| TCGA-AY-6386-01 | 542 | TCGA-D5-6531-01 | 540 | TCGA-G4-6309-01 | 2600 |
| TCGA-AZ-4315-01 | 1776 | TCGA-D5-6532-01 | 555 | TCGA-G4-6310-01 | 1935 |
| TCGA-AZ-4323-01 | 43 | TCGA-D5-6533-01 | 775 | TCGA-G4-6311-01 | 1199 |
| TCGA-AZ-4615-01 | 1002 | TCGA-D5-6534-01 | 1316 | TCGA-G4-6314-01 | 1093 |
| TCGA-AZ-4616-01 | 156 | TCGA-D5-6535-01 | 460 | TCGA-G4-6315-01 | 1883 |
| TCGA-AZ-4681-01 | 3247 | TCGA-D5-6536-01 | 543 | TCGA-G4-6317-01 | 1095 |
| TCGA-AZ-4682-01 | 680 | TCGA-D5-6537-01 | 146 | TCGA-G4-6320-01 | 804 |
| TCGA-AZ-5403-01 | 1910 | TCGA-D5-6538-01 | 521 | TCGA-G4-6321-01 | 672 |
| TCGA-AZ-5407-01 | 2683 | TCGA-D5-6539-01 | 380 | TCGA-G4-6322-01 | 792 |
| TCGA-AZ-6598-01 | 1503 | TCGA-D5-6540-01 | 491 | TCGA-G4-6323-01 | 419 |
| TCGA-AZ-6599-01 | 206 | TCGA-D5-6541-01 | 474 | TCGA-G4-6586-01 | 1089 |
| TCGA-AZ-6601-01 | 3042 | TCGA-D5-6898-01 | 229 | TCGA-G4-6588-01 | 796 |
| TCGA-AZ-6603-01 | 899 | TCGA-D5-6920-01 | 377 | TCGA-G4-6625-01 | 2792 |
| TCGA-AZ-6605-01 | 159 | TCGA-D5-6922-01 | 308 | TCGA-G4-6626-01 | 1422 |
| TCGA-AZ-6607-01 | 97 | TCGA-D5-6924-01 | 435 | TCGA-G4-6628-01 | 2424 |

^a^ Column 1 to 2 represents samples and OS respectively.

**Supplementary Table2. The mutation types in TCGA samples^a^.**

| ***Sample*** | ***Gene*** | ***Mutation Type*** | ***Sample*** | ***Gene*** | ***Muation Type*** |
| --- | --- | --- | --- | --- | --- |
| TCGA-AA-3511-01 | FAM71E2 | INFRAME | TCGA-CM-4746-01 | KIF18B | MISSENSE |
| TCGA-AA-3713-01 | POLG | INFRAME | TCGA-CM-5348-01 | DYNC1I1 | MISSENSE |
| TCGA-AD-6965-01 | FAM71E2 | INFRAME | TCGA-CM-5861-01 | EXO1 | MISSENSE |
| TCGA-AU-6004-01 | FAM71E2 | INFRAME | TCGA-CM-5861-01 | RPL14 | MISSENSE |
| TCGA-CM-4743-01 | CRELD2 | INFRAME | TCGA-CM-5861-01 | SLC5A6 | MISSENSE |
| TCGA-CM-5868-01 | FAM71E2 | INFRAME | TCGA-CM-6161-01 | EXD3 | MISSENSE |
| TCGA-D5-6931-01 | FAM71E2 | INFRAME | TCGA-CM-6161-01 | KLF5 | MISSENSE |
| TCGA-D5-7000-01 | RPL14 | INFRAME | TCGA-CM-6161-01 | TRAPPC5 | MISSENSE |
| TCGA-DM-A285-01 | FAM71E2 | INFRAME | TCGA-CM-6162-01 | EXD3 | MISSENSE |
| TCGA-F4-6463-01 | FAM71E2 | INFRAME | TCGA-CM-6162-01 | EXO1 | MISSENSE |
| TCGA-F4-6569-01 | KLF5 | INFRAME | TCGA-CM-6162-01 | FAM71E2 | MISSENSE |
| TCGA-F4-6703-01 | RPL14 | INFRAME | TCGA-CM-6163-01 | KLF5 | MISSENSE |
| TCGA-F4-6855-01 | FAM71E2 | INFRAME | TCGA-CM-6165-01 | GAD2 | MISSENSE |
| TCGA-G4-6294-01 | POLG | INFRAME | TCGA-CM-6168-01 | PMS1 | MISSENSE |
| TCGA-G4-6298-01 | POLG | INFRAME | TCGA-CM-6168-01 | SLC5A6 | MISSENSE |
| TCGA-G4-6306-01 | SLC5A6 | INFRAME | TCGA-CM-6170-01 | DYNC1I1 | MISSENSE |
| TCGA-G4-6321-01 | POLG | INFRAME | TCGA-CM-6171-01 | HTR2C | MISSENSE |
| TCGA-A6-2675-01 | MACC1 | MISSENSE | TCGA-CM-6171-01 | POLG | MISSENSE |
| TCGA-A6-5661-01 | CAPN6 | MISSENSE | TCGA-CM-6674-01 | CORIN | MISSENSE |
| TCGA-A6-5661-01 | KIF13B | MISSENSE | TCGA-CM-6678-01 | DYNC1I1 | MISSENSE |
| TCGA-A6-5661-01 | NCKIPSD | MISSENSE | TCGA-D5-5540-01 | EXD3 | MISSENSE |
| TCGA-A6-5665-01 | KIF13B | MISSENSE | TCGA-D5-5541-01 | C17orf96 | MISSENSE |
| TCGA-A6-5666-01 | HTR2C | MISSENSE | TCGA-D5-6531-01 | ADAM12 | MISSENSE |
| TCGA-A6-5666-01 | KLF5 | MISSENSE | TCGA-D5-6532-01 | CAPN6 | MISSENSE |
| TCGA-A6-5667-01 | C17orf96 | MISSENSE | TCGA-D5-6533-01 | CAPN6 | MISSENSE |
| TCGA-A6-5667-01 | IRX2 | MISSENSE | TCGA-D5-6534-01 | SLC5A6 | MISSENSE |
| TCGA-A6-5667-01 | RPL14 | MISSENSE | TCGA-D5-6534-01 | ZNF493 | MISSENSE |
| TCGA-A6-6137-01 | PMS1 | MISSENSE | TCGA-D5-6535-01 | HTR2C | MISSENSE |
| TCGA-A6-6141-01 | EXO1 | MISSENSE | TCGA-D5-6535-01 | PMS1 | MISSENSE |
| TCGA-A6-6141-01 | GAD2 | MISSENSE | TCGA-D5-6537-01 | ZNF493 | MISSENSE |
| TCGA-A6-6141-01 | ZNF493 | MISSENSE | TCGA-D5-6540-01 | KIF13B | MISSENSE |
| TCGA-A6-6653-01 | FAM71E2 | MISSENSE | TCGA-D5-6541-01 | EXD3 | MISSENSE |
| TCGA-A6-6653-01 | TMEM8A | MISSENSE | TCGA-D5-6928-01 | TSEN15 | MISSENSE |
| TCGA-A6-6654-01 | GAD2 | MISSENSE | TCGA-D5-6929-01 | TRAPPC5 | MISSENSE |
| TCGA-A6-6781-01 | CORIN | MISSENSE | TCGA-D5-6930-01 | INPP4B | MISSENSE |
| TCGA-A6-6781-01 | PMS1 | MISSENSE | TCGA-D5-6931-01 | CAPN6 | MISSENSE |
| TCGA-AA-3492-01 | KIF5C | MISSENSE | TCGA-D5-6931-01 | GAD2 | MISSENSE |
| TCGA-AA-3492-01 | MACC1 | MISSENSE | TCGA-D5-6931-01 | ZNF493 | MISSENSE |
| TCGA-AA-3662-01 | C17orf96 | MISSENSE | TCGA-D5-7000-01 | EXD3 | MISSENSE |
| TCGA-AA-3662-01 | CAPN6 | MISSENSE | TCGA-D5-7000-01 | GAD2 | MISSENSE |
| TCGA-AA-3662-01 | TRAPPC5 | MISSENSE | TCGA-DM-A0XF-01 | MACC1 | MISSENSE |
| TCGA-AA-3662-01 | TSEN15 | MISSENSE | TCGA-DM-A0XF-01 | RPL14 | MISSENSE |
| TCGA-AA-3663-01 | ARHGEF15 | MISSENSE | TCGA-DM-A1DA-01 | ADAM12 | MISSENSE |
| TCGA-AA-3663-01 | C17orf96 | MISSENSE | TCGA-DM-A1DB-01 | CRELD2 | MISSENSE |
| TCGA-AA-3663-01 | EXD3 | MISSENSE | TCGA-DM-A28F-01 | HTR2C | MISSENSE |
| TCGA-AA-3663-01 | KIF18B | MISSENSE | TCGA-F4-6463-01 | TSEN15 | MISSENSE |
| TCGA-AA-3663-01 | TMEM8A | MISSENSE | TCGA-F4-6570-01 | INPP4B | MISSENSE |
| TCGA-AD-5900-01 | KLKB1 | MISSENSE | TCGA-F4-6703-01 | EXD3 | MISSENSE |
| TCGA-AD-6888-01 | C17orf96 | MISSENSE | TCGA-F4-6703-01 | HTR2C | MISSENSE |
| TCGA-AD-6895-01 | SLC5A6 | MISSENSE | TCGA-F4-6703-01 | SLC5A6 | MISSENSE |
| TCGA-AD-6895-01 | SNTB2 | MISSENSE | TCGA-F4-6703-01 | TRAPPC5 | MISSENSE |
| TCGA-AD-6895-01 | TRAPPC5 | MISSENSE | TCGA-F4-6805-01 | ZNF493 | MISSENSE |
| TCGA-AD-6899-01 | IRX2 | MISSENSE | TCGA-F4-6809-01 | DYNC1I1 | MISSENSE |
| TCGA-AM-5820-01 | ADAM12 | MISSENSE | TCGA-F4-6856-01 | INPP4B | MISSENSE |
| TCGA-AM-5820-01 | EXO1 | MISSENSE | TCGA-G4-6298-01 | TSEN15 | MISSENSE |
| TCGA-AM-5820-01 | HTR2C | MISSENSE | TCGA-G4-6304-01 | RPL14 | MISSENSE |
| TCGA-AM-5821-01 | FAM71E2 | MISSENSE | TCGA-G4-6311-01 | EXD3 | MISSENSE |
| TCGA-AM-5821-01 | KLKB1 | MISSENSE | TCGA-G4-6311-01 | TSEN15 | MISSENSE |
| TCGA-AM-5821-01 | MACC1 | MISSENSE | TCGA-G4-6588-01 | SLC5A6 | MISSENSE |
| TCGA-AM-5821-01 | TRAPPC5 | MISSENSE | TCGA-G4-6588-01 | TRAPPC5 | MISSENSE |
| TCGA-AM-5821-01 | TSEN15 | MISSENSE | TCGA-G4-6626-01 | IRX2 | MISSENSE |
| TCGA-AU-6004-01 | C17orf96 | MISSENSE | TCGA-A6-4105-01 | CRELD2 | TRUNC |
| TCGA-AU-6004-01 | KIF13B | MISSENSE | TCGA-A6-5665-01 | CAPN6 | TRUNC |
| TCGA-AU-6004-01 | TNRC6C | MISSENSE | TCGA-A6-5665-01 | KLKB1 | TRUNC |
| TCGA-AY-6197-01 | C17orf96 | MISSENSE | TCGA-A6-5665-01 | POLG | TRUNC |
| TCGA-AY-6386-01 | TRAPPC5 | MISSENSE | TCGA-A6-6140-01 | RGMB | TRUNC |
| TCGA-AZ-4315-01 | ADAM12 | MISSENSE | TCGA-A6-6141-01 | CRELD2 | TRUNC |
| TCGA-AZ-4315-01 | ARHGEF15 | MISSENSE | TCGA-A6-6781-01 | CORIN | TRUNC |
| TCGA-AZ-4315-01 | CORIN | MISSENSE | TCGA-AA-3492-01 | TNRC6C | TRUNC |
| TCGA-AZ-4315-01 | KIF13B | MISSENSE | TCGA-AD-5900-01 | ARHGEF15 | TRUNC |
| TCGA-AZ-4315-01 | KIF18B | MISSENSE | TCGA-AD-5900-01 | INPP4B | TRUNC |
| TCGA-AZ-4315-01 | PMS1 | MISSENSE | TCGA-AD-6890-01 | SNTB2 | TRUNC |
| TCGA-AZ-4315-01 | SNTB2 | MISSENSE | TCGA-AD-6964-01 | DYNC1I1 | TRUNC |
| TCGA-AZ-4315-01 | ZNF493 | MISSENSE | TCGA-AU-6004-01 | INPP4B | TRUNC |
| TCGA-AZ-4615-01 | EXD3 | MISSENSE | TCGA-AY-6197-01 | TNRC6C | TRUNC |
| TCGA-AZ-4615-01 | KLKB1 | MISSENSE | TCGA-AY-6386-01 | CRELD2 | TRUNC |
| TCGA-AZ-6598-01 | POLG | MISSENSE | TCGA-AY-6386-01 | RGMB | TRUNC |
| TCGA-AZ-6599-01 | KLF5 | MISSENSE | TCGA-AZ-6598-01 | ARHGEF15 | TRUNC |
| TCGA-AZ-6603-01 | TRAPPC5 | MISSENSE | TCGA-AZ-6598-01 | FAM71E2 | TRUNC |
| TCGA-CA-6717-01 | CAPN6 | MISSENSE | TCGA-AZ-6598-01 | KIF18B | TRUNC |
| TCGA-CA-6717-01 | EXO1 | MISSENSE | TCGA-AZ-6598-01 | MACC1 | TRUNC |
| TCGA-CA-6717-01 | HTR2C | MISSENSE | TCGA-CA-6717-01 | CAPN6 | TRUNC |
| TCGA-CA-6717-01 | KIF13B | MISSENSE | TCGA-CA-6717-01 | PMS1 | TRUNC |
| TCGA-CA-6717-01 | KIF5C | MISSENSE | TCGA-CA-6717-01 | SNTB2 | TRUNC |
| TCGA-CA-6717-01 | NCKIPSD | MISSENSE | TCGA-CA-6718-01 | KIF18B | TRUNC |
| TCGA-CA-6717-01 | TNRC6C | MISSENSE | TCGA-CM-4743-01 | EXO1 | TRUNC |
| TCGA-CA-6718-01 | ARHGEF15 | MISSENSE | TCGA-CM-4746-01 | CORIN | TRUNC |
| TCGA-CA-6718-01 | FAM71E2 | MISSENSE | TCGA-CM-5861-01 | ARHGEF15 | TRUNC |
| TCGA-CA-6718-01 | KIF13B | MISSENSE | TCGA-CM-6169-01 | CRELD2 | TRUNC |
| TCGA-CA-6718-01 | SNTB2 | MISSENSE | TCGA-CM-6171-01 | KLKB1 | TRUNC |
| TCGA-CA-6718-01 | TSEN15 | MISSENSE | TCGA-D5-6540-01 | CRELD2 | TRUNC |
| TCGA-CK-4952-01 | TRAPPC5 | MISSENSE | TCGA-D5-6924-01 | RGMB | TRUNC |
| TCGA-CK-5913-01 | TNRC6C | MISSENSE | TCGA-D5-6932-01 | RGMB | TRUNC |
| TCGA-CK-5916-01 | ARHGEF15 | MISSENSE | TCGA-DM-A285-01 | PMS1 | TRUNC |
| TCGA-CK-5916-01 | CAPN6 | MISSENSE | TCGA-G4-6295-01 | TMEM8A | TRUNC |
| TCGA-CK-5916-01 | TMEM8A | MISSENSE | TCGA-G4-6323-01 | ADAM12 | TRUNC |
| TCGA-CM-4743-01 | GAD2 | MISSENSE | TCGA-G4-6586-01 | KIF5C | TRUNC |
| TCGA-CM-4743-01 | KLKB1 | MISSENSE | TCGA-G4-6588-01 | EXD3 | TRUNC |
| TCGA-CM-4746-01 | KIF13B | MISSENSE | TCGA-G4-6588-01 | KLKB1 | TRUNC |

^a^ Columns 1 to 3 represents the samples, mutaion gene and mutaion type respectively.

**Supplementary Table 3. The corresponding genes of prognosis-related features.**

**Supplementary Table 3a. The corresponding genes of prognosis-related exon^a^.**

| ***prognosis-related exon*** | ***gene*** | ***prognosis-related exon*** | ***gene*** |
| --- | --- | --- | --- |
| chr1:179354373-179354494:+ | AXDND1 | chr2:106226785-106227016:- | LOC285000 |
| chr1:29315868-29315947:+ | EPB41 | chr2:201642472-201642537:+ | AOX2P |
| chr1:40126751-40126935:- | NT5C1A | chr2:27479254-27479388:- | SLC30A3 |
| chr1:70339150-70340687:+ |  | chr2:86118299-86118333:+ |  |
| chr1:74547602-74549900:- |  | chr20:31494368-31494435:+ |  |
| chr10:48265297-48265392:+ | ANXA8L1 | chr20:36977951-36978065:+ | LBP |
| chr10:48265297-48265392:+ | ANXA8 | chr3:153220192-153220483:+ | C3orf79 |
| chr10:88414314-88414684:+ | OPN4 | chr4:119145646-119145828:+ | NDST3 |
| chr11:120132422-120132468:+ |  | chr4:25674709-25674876:+ | SLC34A2 |
| chr12:45408539-45410401:- | DBX2 | chr5:152351826-152351909:- |  |
| chr14:106657703-106657713:- |  | chr5:175523827-175523903:+ | FAM153B |
| chr14:20801413-20801457:- | CCNB1IP1 | chr5:90074682-90074914:+ | GPR98 |
| chr14:94731724-94731810:+ | PPP4R4 | chr6:10959017-10959108:+ | SYCP2L |
| chr14:94732131-94732224:+ | PPP4R4 | chr6:152734487-152734666:- | SYNE1 |
| chr14:95107638-95108237:+ | SERPINA13P | chr7:12420112-12420359:- | VWDE |
| chr15:66311735-66312097:- |  | chr7:127894457-127897682:+ | LEP |
| chr16:77396002-77396161:- | ADAMTS18 | chr7:129948147-129948237:+ | CPA4 |
| chr17:10426407-10426502:- | MYH2 | chr7:142723287-142724219:- | OR9A2 |
| chr17:66985168-66985259:- | ABCA9 | chr8:143570697-143570799:+ | BAI1 |
| chr19:14693896-14694015:+ | CLEC17A | chrY:2709623-2709668:+ | RPS4Y1 |

^a^ Columns 1 to 2 represents the prognosis-related exons and corresponding genes.

**Supplementary Table 3b. The corresponding genes of prognosis-related methylations^a^.**

| ***prognosis-related methylation*** | ***gene*** | ***prognosis-related methylation*** | ***gene*** |
| --- | --- | --- | --- |
| cg01102158 | TSSC1 | cg12091396 | C10orf35 |
| cg02009256 | GLS2 | cg13883256 | FAM174A |
| cg02654360 | PDE3B | cg15844835 | FCHSD1 |
| cg02654360 | PSMA1 | cg18421529 | MRAS |
| cg02760766 | CELF6 | cg19585103 | SKAP1 |
| cg02760766 | C15orf34 | cg20717205 | ZNF35 |
| cg02760766 | HEXA | cg22267597 | MYH10 |
| cg04096096 | TRAPPC2 | cg22346124 | ASCL2 |
| cg04096096 | OFD1 | cg23835677 | FGFR3 |
| cg04353251 | MIR196A1 | cg25074185 | PHOX2A |
| cg06671690 | RNF7 | cg26151087 | ZNF493 |
| cg06685724 | C7orf46 | cg27525037 | GLUL |
| cg11027354 | DAP | cg27624313 | GTF2IRD1 |

^a^ Columns 1 to 2 represents the prognosis-related methylations and corresponding genes.

**Supplementary Table 4. Corresponding genes of prognosis-related features in quadruple-omics^a^.**

| ***gene*** | ***class*** | ***PPI color*** | ***gene*** | ***class*** | ***PPI color*** |
| --- | --- | --- | --- | --- | --- |
| MRAP | gene | green | OFD1 | methylation | orange |
| KRTDAP | gene | green | MIR196A1 | methylation | orange |
| OR2T5 | gene | green | RNF7 | methylation | orange |
| OR4N2 | gene | green | C7orf46 | methylation | orange |
| LECT2 | gene | green | DAP | methylation | orange |
| LIPK | gene | green | C10orf35 | methylation | orange |
| TCF23 | gene | green | FAM174A | methylation | orange |
| SFTPA1 | gene | green | FCHSD1 | methylation | orange |
| C20orf186 | gene | green | MRAS | methylation | orange |
| OR52N4 | gene | green | SKAP1 | methylation | orange |
| SLC22A8 | gene | green | ZNF35 | methylation | orange |
| MYH2 | gene_exon | lightblue | MYH10 | methylation | orange |
| ABCA9 | exon | blue | ASCL2 | methylation | orange |
| ADAMTS18 | exon | blue | FGFR3 | methylation | orange |
| ANXA8 | exon | blue | PHOX2A | methylation | orange |
| ANXA8L1 | exon | blue | ZNF493 | methylation_mutation | pink |
| AOX2P | exon | blue | GLUL | methylation | orange |
| AXDND1 | exon | blue | GTF2IRD1 | methylation | orange |
| BAI1 | exon | blue | KLF5 | mutation | red |
| C3orf79 | exon | blue | SNTB2 | mutation | red |
| CCNB1IP1 | exon | blue | ADAM12 | mutation | red |
| CLEC17A | exon | blue | TRAPPC5 | mutation | red |
| CPA4 | exon | blue | HTR2C | mutation | red |
| DBX2 | exon | blue | C17orf96 | mutation | red |
| EPB41 | exon | blue | RGMB | mutation | red |
| FAM153B | exon | blue | MACC1 | mutation | red |
| GPR98 | exon | blue | TSEN15 | mutation | red |
| LBP | exon | blue | PMS1 | mutation | red |
| LEP | exon | blue | TNRC6C | mutation | red |
| LOC285000 | exon | blue | SLC5A6 | mutation | red |
| NDST3 | exon | blue | ARHGEF15 | mutation | red |
| NT5C1A | exon | blue | EXD3 | mutation | red |
| OPN4 | exon | blue | RPL14 | mutation | red |
| OR9A2 | exon | blue | POLG | mutation | red |
| PPP4R4 | exon | blue | CAPN6 | mutation | red |
| RPS4Y1 | exon | blue | DYNC1I1 | mutation | red |
| SERPINA13P | exon | blue | IRX2 | mutation | red |
| SLC30A3 | exon | blue | KIF13B | mutation | red |
| SLC34A2 | exon | blue | INPP4B | mutation | red |
| SYCP2L | exon | blue | CRELD2 | mutation | red |
| SYNE1 | exon | blue | GAD2 | mutation | red |
| VWDE | exon | blue | EXO1 | mutation | red |
| TSSC1 | methylation | orange | KIF5C | mutation | red |
| GLS2 | methylation | orange | FAM71E2 | mutation | red |
| PDE3B | methylation | orange | KLKB1 | mutation | red |
| PSMA1 | methylation | orange | TMEM8A | mutation | red |
| CELF6 | methylation | orange | KIF18B | mutation | red |
| C15orf34 | methylation | orange | NCKIPSD | mutation | red |
| HEXA | methylation | orange | CORIN | mutation | red |
| TRAPPC2 | methylation | orange |  |  |  |

^a^ Columns 1 to 3 represents the corresponding gene, omics data classes and node color of PPI network in Figure 3a.

**Supplementary Table 5. The information of prognosis-telated features^a^.**

| ***feature*** | ***class*** | ***abbreviation*** | ***color*** | ***node_size*** |
| --- | --- | --- | --- | --- |
| MRAP | gene | g1 | green | 0 |
| KRTDAP | gene | g2 | green | 0 |
| OR2T5 | gene | g3 | green | 0 |
| OR4N2 | gene | g4 | green | 0 |
| LECT2 | gene | g5 | green | 1 |
| LIPK | gene | g6 | green | 0 |
| TCF23 | gene | g7 | green | 0 |
| SFTPA1 | gene | g8 | green | 0 |
| C20orf186 | gene | g9 | green | 0 |
| OR52N4 | gene | g10 | green | 0 |
| SLC22A8 | gene | g11 | green | 0 |
| MYH2 | gene | g12 | green | 0 |
| chr1:179354373-179354494:+ | exon | e1 | blue | 0 |
| chr1:29315868-29315947:+ | exon | e2 | blue | 0 |
| chr1:40126751-40126935:- | exon | e3 | blue | 0 |
| chr1:70339150-70340687:+ | exon | e4 | blue | 0 |
| chr1:74547602-74549900:- | exon | e5 | blue | 0 |
| chr10:48265297-48265392:+ | exon | e6 | blue | 0 |
| chr10:88414314-88414684:+ | exon | e7 | blue | 0 |
| chr11:120132422-120132468:+ | exon | e8 | blue | 1 |
| chr12:45408539-45410401:- | exon | e9 | blue | 0 |
| chr14:106657703-106657713:- | exon | e10 | blue | 0 |
| chr14:20801413-20801457:- | exon | e11 | blue | 0 |
| chr14:94731724-94731810:+ | exon | e12 | blue | 0 |
| chr14:94732131-94732224:+ | exon | e13 | blue | 0 |
| chr14:95107638-95108237:+ | exon | e14 | blue | 0 |
| chr15:66311735-66312097:- | exon | e15 | blue | 0 |
| chr16:77396002-77396161:- | exon | e16 | blue | 0 |
| chr17:10426407-10426502:- | exon | e17 | blue | 0 |
| chr17:66985168-66985259:- | exon | e18 | blue | 1 |
| chr19:14693896-14694015:+ | exon | e19 | blue | 0 |
| chr2:106226785-106227016:- | exon | e20 | blue | 2 |
| chr2:201642472-201642537:+ | exon | e21 | blue | 0 |
| chr2:27479254-27479388:- | exon | e22 | blue | 1 |
| chr2:86118299-86118333:+ | exon | e23 | blue | 1 |
| chr20:31494368-31494435:+ | exon | e24 | blue | 0 |
| chr20:36977951-36978065:+ | exon | e25 | blue | 2 |
| chr3:153220192-153220483:+ | exon | e26 | blue | 0 |
| chr4:119145646-119145828:+ | exon | e27 | blue | 0 |
| chr4:25674709-25674876:+ | exon | e28 | blue | 1 |
| chr5:152351826-152351909:- | exon | e29 | blue | 0 |
| chr5:175523827-175523903:+ | exon | e30 | blue | 0 |
| chr5:90074682-90074914:+ | exon | e31 | blue | 0 |
| chr6:10959017-10959108:+ | exon | e32 | blue | 1 |
| chr6:152734487-152734666:- | exon | e33 | blue | 0 |
| chr7:12420112-12420359:- | exon | e34 | blue | 0 |
| chr7:127894457-127897682:+ | exon | e35 | blue | 0 |
| chr7:129948147-129948237:+ | exon | e36 | blue | 0 |
| chr7:142723287-142724219:- | exon | e37 | blue | 0 |
| chr8:143570697-143570799:+ | exon | e38 | blue | 0 |
| chrY:2709623-2709668:+ | exon | e39 | blue | 0 |
| cg13883256 | methylation | me1 | orange | 0 |
| cg04353251 | methylation | me2 | orange | 0 |
| cg02009256 | methylation | me3 | orange | 0 |
| cg01102158 | methylation | me4 | orange | 0 |
| cg22346124 | methylation | me5 | orange | 0 |
| cg27525037 | methylation | me6 | orange | 0 |
| cg18421529 | methylation | me7 | orange | 0 |
| cg06685724 | methylation | me8 | orange | 2 |
| cg02654360 | methylation | me9 | orange | 0 |
| cg27624313 | methylation | me10 | orange | 0 |
| cg26151087 | methylation | me11 | orange | 0 |
| cg12091396 | methylation | me12 | orange | 0 |
| cg02760766 | methylation | me13 | orange | 0 |
| cg11027354 | methylation | me14 | orange | 0 |
| cg22267597 | methylation | me15 | orange | 0 |
| cg06671690 | methylation | me16 | orange | 0 |
| cg25074185 | methylation | me17 | orange | 0 |
| cg15844835 | methylation | me18 | orange | 0 |
| cg23835677 | methylation | me19 | orange | 0 |
| cg04096096 | methylation | me20 | orange | 1 |
| cg20717205 | methylation | me21 | orange | 1 |
| cg19585103 | methylation | me22 | orange | 0 |
| KLF5 | mutation | mu1 | red | 0 |
| SNTB2 | mutation | mu2 | red | 0 |
| ADAM12 | mutation | mu3 | red | 0 |
| TRAPPC5 | mutation | mu4 | red | 0 |
| HTR2C | mutation | mu5 | red | 0 |
| C17orf96 | mutation | mu6 | red | 0 |
| RGMB | mutation | mu7 | red | 0 |
| MACC1 | mutation | mu8 | red | 0 |
| TSEN15 | mutation | mu9 | red | 0 |
| PMS1 | mutation | mu10 | red | 0 |
| TNRC6C | mutation | mu11 | red | 0 |
| SLC5A6 | mutaion | mu12 | red | 0 |
| ARHGEF15 | mutation | mu13 | red | 0 |
| EXD3 | mutation | mu14 | red | 0 |
| RPL14 | mutation | mu15 | red | 0 |
| POLG | mutation | mu16 | red | 0 |
| CAPN6 | mutation | mu17 | red | 0 |
| DYNC1I1 | mutation | mu18 | red | 0 |
| IRX2 | mutation | mu19 | red | 0 |
| KIF13B | mutation | mu20 | red | 0 |
| INPP4B | mutation | mu21 | red | 0 |
| CRELD2 | mutation | mu22 | red | 0 |
| GAD2 | mutation | mu23 | red | 0 |
| EXO1 | mutation | mu24 | red | 0 |
| KIF5C | mutation | mu25 | red | 0 |
| FAM71E2 | mutation | mu26 | red | 0 |
| KLKB1 | mutation | mu27 | red | 0 |
| TMEM8A | mutation | mu28 | red | 0 |
| KIF18B | mutation | mu29 | red | 0 |
| NCKIPSD | mutation | mu30 | red | 0 |
| ZNF493 | mutation | mu31 | red | 0 |
| CORIN | mutation | mu32 | red | 0 |

^a^ Columns 1 to 5 represents the features, corresponding omics data classes and abbreviations,

color, and node size which illustrated in Figure 5 respectively.

**Supplementary Table 6. The differentially expressed PRBs from single cell-based expression ^a^.**

|  | ***all cell FPKM*** | | ***all cell count*** | | ***epithelial cell FPKM*** | | ***epithelial cell count*** | |
| --- | --- | --- | --- | --- | --- | --- | --- | --- |
| **gene** | **P-value** | **FC** | **P-value** | **FC** | **P-value** | **FC** | **P-value** | **FC** |
| EPB41 | 5.25E-01 | 1.15E+00 | 5.29E-01 | 2.31E+00 | 9.16E-01 | 1.12E+00 | 8.88E-01 | 2.17E+00 |
| PSMA1 | 7.23E-03 | 1.27E+00 | 2.81E-03 | 9.74E-01 | 1.05E-02 | 1.42E+00 | 1.92E-02 | 1.02E+00 |
| FGFR3 | 7.42E-09 | 1.68E-01 | 2.31E-07 | 1.15E-01 | 7.15E-08 | 1.72E-01 | 7.48E-08 | 1.05E-01 |
| MRAS | 9.86E-01 | 5.86E+00 | 7.20E-01 | 1.05E+02 | 6.20E-01 | 9.04E-01 | 6.19E-01 | 1.76E+00 |
| LEP | 8.73E-01 | 1.36E+00 | 6.85E-01 | 1.82E+00 | 1.24E-01 | Inf | 1.24E-01 | Inf |
| C7orf46 | 9.73E-02 | 1.32E+00 | 1.28E-02 | 1.75E+00 | 3.14E-02 | 2.33E+00 | 3.17E-02 | 2.72E+00 |
| LOC285000 | 1.37E-01 | 2.09E+00 | 6.21E-02 | 6.15E-01 | 7.12E-02 | 3.17E+01 | 7.14E-02 | 1.71E+01 |
| LBP | 4.85E-01 | 2.77E-01 | 6.73E-01 | 1.18E+00 | 8.89E-01 | 1.19E+00 | 8.95E-01 | 1.96E+00 |
| LECT2 | 2.85E-01 | Inf | 7.74E-01 | 4.26E+00 | 2.79E-01 | Inf | 2.79E-01 | Inf |
| SLC30A3 | 8.35E-01 | 1.77E+01 | 6.10E-01 | 1.09E+02 | 4.70E-01 | 2.33E+01 | 4.72E-01 | 1.35E+02 |
| RNF7 | 3.35E-01 | 1.01E+00 | 1.16E-01 | 1.20E+00 | 8.93E-13 | 2.57E+01 | 1.04E-01 | 9.56E-01 |
| ZNF35 | 1.33E-01 | 2.61E+00 | 7.47E-03 | 3.16E-01 | 1.88E-02 | 2.03E+02 | 1.97E-01 | 2.78E+00 |
| DYNC1I1 | 2.32E-01 | 8.13E-02 | 3.92E-01 | 2.12E-01 | 8.99E-01 | 3.80E+00 | 8.99E-01 | 7.06E+00 |

^a^ Columns 1 represents the PRBs detected in this study. And Columns 2 to 3, 4 to 5, 6 to 7, 8 to 9 stands for the P value and FC of PRBs in tumor and normal cell from corresponding dataset.

**Supplementary Table 7. The risk evaluation of clinical features by the Cox PH model ^a^**.

| ***Clinical Features*** | ***Univariate Analysis*** | |
| --- | --- | --- |
|  | ***HR (95% CI)*** | ***P value*** |
| age | 1.027 (1.011-1.042) | 0.000824 *** |
| gender | 0.941 (0.658-1.346) | 0.741 |
| weight | 0.984 (0.969-0.998) | 0.0252 * |
| histological type | 1.457 (0.9002-2.358) | 0.126 |
| history of colon polyps | 0.728 (0.454-1.166) | 0.186 |
| lymphatic invasion | 2.083 (1.417-3.061) | 0.000189 *** |
| person neoplasm cancer status | 6.465 (4.250-9.836) | <2e-16 *** |
| pathologic stage | 2.005 (1.626-2.472) | 7.91e-11 *** |
| pathologic T | 2.559 (1.773-3.693) | 5.17e-07 *** |
| number of first degree relatives with cancer diagnosis | 0.719 (0.422-1.225) | 0.224 |
| venous invasion | 2.204 (1.477-3.290) | 0.00011 *** |

^a^ Columns 1 represents the possible prognosis-related clinical features. And Columns 2 to 3 represents hazard ratio (HR), 95% Confidence Interval (CI) and significance (P value) of Univariate Analysis.

*p<0.05, **p<0.01, ***p<0.001

**Supplementary Table 8. The counts for different sample groups ^a^.**

| ***clinical feature*** | ***group*** | ***all counts*** | ***positive counts*** | ***negative counts*** |
| --- | --- | --- | --- | --- |
| age | >65 | 111 | 20 | 91 |
|  | <=65 | 91 | 11 | 80 |
| weight | <80 | 85 | 15 | 70 |
|  | >=80 | 85 | 8 | 77 |
| lymphatic invasion | Yes | 49 | 5 | 44 |
|  | No | 132 | 22 | 110 |
| person neoplasm cancer status | Free | 135 | 14 | 121 |
|  | With | 45 | 5 | 40 |
| pathologic stage | <3 | 111 | 20 | 91 |
|  | >=3 | 82 | 11 | 71 |
| pathologic T stage | <3 | 33 | 2 | 31 |
|  | >=3 | 169 | 29 | 140 |
| number of first degree relatives with cancer diagnosis | 0 | 130 | 22 | 108 |
|  | >0 | 25 | 5 | 20 |
| venous invasion | Yes | 38 | 5 | 33 |
|  | No | 140 | 22 | 118 |

^a^ Columns 1 represents the prognosis-related clinical features. And Columns 2 to 5 represents the sample group, all counts, positive counts, negative counts of samples in each group.

**Supplementary Table 9. The significant PRBs for different sample groups ^a^.**

| *clinical feature* | *sample group* | *marker* | *P-value* |
| --- | --- | --- | --- |
| age | >65 | SLC30A3 | 0.027 |
|  |  | LOC285000 | 0.013 |
|  |  | LEP | 0.00029 |
|  | <=65 | SLC30A3 | 0.023 |
|  |  | DYNC1I1 | 0.00038 |
| weight | <80 | SLC30A3 | 0.0067 |
|  |  | DYNC1I1 | 0.031 |
|  | >=80 | SLC30A3 | 0.015 |
|  |  | LEP | 0.009 |
|  |  | DYNC1I1 | 0.046 |
|  |  | LECT2 | 0.013 |
| lymphatic invasion | Yes | RNF7 | 0.025 |
|  |  | MRAS | 0.018 |
|  | No | LEP | 0.013 |
|  |  | LECT2 | 0.004 |
| person neoplasm cancer status | Free | LOC285000 | 0.02 |
|  |  | LEP | 0.008 |
|  | With | SLC30A3 | 0.023 |
|  |  | DYNC1I1 | 0.011 |
| pathologic stage | <3 | SLC30A3 | 0.015 |
|  |  | LOC285000 | 0.039 |
|  |  | LEP | 0.031 |
|  | >=3 | MRAS | 0.044 |
|  |  | LEP | 0.00089 |
|  |  | LECT2 | 0.033 |
| pathologic T stage | >=3 | RNF7 | 0.014 |
|  |  | SLC30A3 | 0.016 |
|  |  | LEP | 0.00013 |
|  |  | DYNC1I1 | 0.04 |
|  |  | LECT2 | 0.0093 |
| number of first degree relatives with cancer diagnosis | =0 | SLC30A3 | 0.0014 |
|  |  | LOC285000 | 0.019 |
|  |  | LEP | 0.00086 |
|  |  | LECT2 | 0.0041 |
|  | >0 | DYNC1I1 | 0.00091 |
| venous invasion | Yes | RNF7 | 0.02 |
|  |  | MRAS | 0.005 |
|  |  | SLC30A3 | 0.031 |
|  |  | LEP | 0.018 |
|  | No | LEP | 0.023 |
|  |  | LECT2 | 0.03 |

^a^ Columns 1 represents the prognosis-related clinical features. And Columns 2 stands for the sample groups. Column 3 and 4 stands for the significant PRBs and their corresponding p values by log-rank test.

**Supplementary Table 10. The risk evalution of prognosis-related features by the Cox PH model ^a^.**

|  |  | **Univariate Analysis^$^** | | **Multivariate Analysis^$^** | |
| --- | --- | --- | --- | --- | --- |
| **feature** | **gene** | **HR** | **P value** | **HR** | **P value** |
| C20orf186 | C20orf186 | 1.385 | 0.0131 * | 0.2694 | 0.007725 ** |
| KRTDAP | KRTDAP | 1.075 | 0.744 |  |  |
| LECT2 | LECT2 | 1.584 | 0.06 |  |  |
| LIPK | LIPK | 1.795 | 0.0278 * | 0.2192 | 0.082239 |
| MRAP | MRAP | 1.319 | 0.00383 ** | 0.2303 | 0.007475 ** |
| MYH2 | MYH2 | 1.787 | 0.0246 * | 0.9215 | 0.92016 |
| OR2T5 | OR2T5 | 1.354 | 0.604 |  |  |
| OR4N2 | OR4N2 | 4.711 | 0.0264 * | 1.549 | 0.660733 |
| OR52N4 | OR52N4 | 1.264 | 0.445 |  |  |
| SFTPA1 | SFTPA1 | 1.014 | 0.919 |  |  |
| SLC22A8 | SLC22A8 | 30.18 | 0.000166 *** | 181.6 | 0.009125 ** |
| TCF23 | TCF23 | 1.732 | 0.00154 ** | 2.163 | 0.116016 |
| chr1:179354373-179354494:+ | AXDND1 | 4.211 | 0.788 |  |  |
| chr1:29315868-29315947:+ | EPB41 | 10.11 | 0.678 |  |  |
| chr1:40126751-40126935:- | NT5C1A | 32154 | 0.00118 ** | 2.46E-07 | 0.215199 |
| chr1:70339150-70340687:+ |  | 2748655 | 0.0248 * | 52.85 | 0.782598 |
| chr1:74547602-74549900:- |  | 2.80E+58 | 0.011 * | 3.03E+25 | 0.637834 |
| chr10:48265297-48265392:+ | ANXA8L1/ANXA8 | 1.689 | 0.0043 ** | 3.164 | 0.074217 |
| chr10:88414314-88414684:+ | OPN4 | 0.0005781 | 0.611 |  |  |
| chr11:120132422-120132468:+ |  | 2 | 0.702 |  |  |
| chr12:45408539-45410401:- | DBX2 | 135320369 | 0.00118 ** | 1.30E+21 | 0.048032 * |
| chr14:106657703-106657713:- |  | 1.206 | 0.733 |  |  |
| chr14:20801413-20801457:- | CCNB1IP1 | 1.232 | 0.0708 |  |  |
| chr14:94731724-94731810:+ | PPP4R4 | 2.423 | 0.0107 * | 0.4453 | 0.70208 |
| chr14:94732131-94732224:+ | PPP4R4 | 2.619 | 0.00725 ** | 6.651 | 0.426228 |
| chr14:95107638-95108237:+ | SERPINA13P | 0 | 0.997 |  |  |
| chr15:66311735-66312097:- |  | 99.2 | 0.11 |  |  |
| chr16:77396002-77396161:- | ADAMTS18 | 7.32 | 0.000874 *** | 0.6218 | 0.769252 |
| chr17:10426407-10426502:- | MYH2 | 92155 | 0.000711 *** | 0.3012 | 0.919158 |
| chr17:66985168-66985259:- | ABCA9 | 3.235 | 0.00339 ** | 1.447 | 0.787987 |
| chr19:14693896-14694015:+ | CLEC17A | 7.396 | 0.000306 *** | 13.97 | 0.117757 |
| chr2:106226785-106227016:- | LOC285000 | 22.2 | 0.0195 * | 15.94 | 0.251326 |
| chr2:201642472-201642537:+ | AOX2P | 376.3 | 0.0212 * | 1.16E+08 | 0.000229 *** |
| chr2:27479254-27479388:- | SLC30A3 | 11.67 | 1.52e-05 *** | 10.68 | 0.089687 |
| chr2:86118299-86118333:+ |  | 7.49 | 0.0748 |  |  |
| chr20:31494368-31494435:+ |  | 15.31 | 0.00775 ** | 171.2 | 0.010245 * |
| chr20:36977951-36978065:+ | LBP | 1.759 | 0.00405 ** | 4.674 | 0.024884 * |
| chr3:153220192-153220483:+ | C3orf79 | 0.5658 | 0.962 |  |  |
| chr4:119145646-119145828:+ | NDST3 | 0.8549 | 0.946 |  |  |
| chr4:25674709-25674876:+ | SLC34A2 | 1.161 | 0.663 |  |  |
| chr5:152351826-152351909:- |  | 2.93E-69 | 0.996 |  |  |
| chr5:175523827-175523903:+ | FAM153B | 2406 | 0.00875 ** | 8.717 | 0.724952 |
| chr5:90074682-90074914:+ | GPR98 | 1.651 | 0.523 |  |  |
| chr6:10959017-10959108:+ | SYCP2L | 2.574 | 0.0793 |  |  |
| chr6:152734487-152734666:- | SYNE1 | 1014528 | 0.00256 ** | 2.08E+11 | 0.023539 * |
| chr7:12420112-12420359:- | VWDE | 4.363 | 0.00093 *** | 0.3806 | 0.339601 |
| chr7:127894457-127897682:+ | LEP | 1.872 | 0.006 ** | 2.198 | 0.431435 |
| chr7:129948147-129948237:+ | CPA4 | 3.489 | 1.08e-05 *** | 26.23 | 0.004817 ** |
| chr7:142723287-142724219:- | OR9A2 | 841862604 | 0.128 |  |  |
| chr8:143570697-143570799:+ | BAI1 | 7.481 | 0.00184 ** | 0.09774 | 0.175161 |
| chrY:2709623-2709668:+ | RPS4Y1 | 1.125 | 0.166 |  |  |
| cg01102158 | TSSC1 | 672.3 | 0.0297 * | 5059000 | 0.001411 ** |
| cg02009256 | GLS2 | 3.131 | 0.307 |  |  |
| cg02654360 | PDE3B/PSMA1 | 25.08 | 0.077 |  |  |
| cg02760766 | CELF6/C15orf34/HEXA | 5.886 | 0.567 |  |  |
| cg04096096 | TRAPPC2/OFD1 | 3.77 | 0.667 |  |  |
| cg04353251 | MIR196A1 | 8.115 | 0.0235 * | 0.05817 | 0.320404 |
| cg06671690 | RNF7 | 27.74 | 0.0234 * | 6008 | 0.002395 ** |
| cg06685724 | C7orf46 | 1.752 | 0.552 |  |  |
| cg11027354 | DAP | 7.103 | 0.397 |  |  |
| cg12091396 | C10orf35 | 32.85 | 0.0302 * | 0.1721 | 0.690846 |
| cg13883256 | FAM174A | 3.368 | 0.399 |  |  |
| cg15844835 | FCHSD1 | 0.4863 | 0.749 |  |  |
| cg18421529 | MRAS | 10.39 | 0.201 |  |  |
| cg19585103 | SKAP1 | 12.05 | 0.0954 |  |  |
| cg20717205 | ZNF35 | 21.4 | 0.0222 * | 7.174 | 0.471894 |
| cg22267597 | MYH10 | 0.625 | 0.862 |  |  |
| cg22346124 | ASCL2 | 6.504 | 0.201 |  |  |
| cg23835677 | FGFR3 | 1.271 | 0.851 |  |  |
| cg25074185 | PHOX2A | 22.92 | 0.0172 * | 0.5902 | 0.839744 |
| cg26151087 | ZNF493 | 0.8657 | 0.945 |  |  |
| cg27525037 | GLUL | 108.4 | 0.0035 ** | 755.2 | 0.029246 * |
| cg27624313 | GTF2IRD1 | 0.7439 | 0.853 |  |  |
| ADAM12 | ADAM12 | 0.9013 | 0.918 |  |  |
| ARHGEF15 | ARHGEF15 | 2.274 | 0.172 |  |  |
| C17orf96 | C17orf96 | 0.8672 | 0.888 |  |  |
| CAPN6 | CAPN6 | 0.9786 | 0.983 |  |  |
| CORIN | CORIN | 1.08E-07 | 0.996 |  |  |
| CRELD2 | CRELD2 | 2.281 | 0.26 |  |  |
| DYNC1I1 | DYNC1I1 | 2.791 | 0.0896 |  |  |
| EXD3 | EXD3 | 3.82E-08 | 0.996 |  |  |
| EXO1 | EXO1 | 3.519 | 0.0897 |  |  |
| FAM71E2 | FAM71E2 | 1.625 | 0.421 |  |  |
| GAD2 | GAD2 | 1.07E-07 | 0.996 |  |  |
| HTR2C | HTR2C | 0.9106 | 0.926 |  |  |
| INPP4B | INPP4B | 2.44 | 0.224 |  |  |
| IRX2 | IRX2 | 1.916 | 0.372 |  |  |
| KIF13B | KIF13B | 0.6325 | 0.651 |  |  |
| KIF18B | KIF18B | 1.864 | 0.392 |  |  |
| KIF5C | KIF5C | 1.649 | 0.491 |  |  |
| KLF5 | KLF5 | 2.193 | 0.284 |  |  |
| KLKB1 | KLKB1 | 1.07E-07 | 0.996 |  |  |
| MACC1 | MACC1 | 3.938 | 0.00988 ** | 22.87 | 0.000337 *** |
| NCKIPSD | NCKIPSD | 1.873 | 0.389 |  |  |
| PMS1 | PMS1 | 0.7965 | 0.823 |  |  |
| POLG | POLG | 2.002 | 0.251 |  |  |
| RGMB | RGMB | 3.211 | 0.115 |  |  |
| RPL14 | RPL14 | 1.497 | 0.579 |  |  |
| SLC5A6 | SLC5A6 | 3.87E-08 | 0.997 |  |  |
| SNTB2 | SNTB2 | 1.481 | 0.589 |  |  |
| TMEM8A | TMEM8A | 1.185 | 0.868 |  |  |
| TNRC6C | TNRC6C | 0.8156 | 0.841 |  |  |
| TRAPPC5 | TRAPPC5 | 0.7066 | 0.732 |  |  |
| TSEN15 | TSEN15 | 3.449 | 0.0416 * | 3.612 | 0.180724 |
| ZNF493 | ZNF493 | 0.8163 | 0.841 |  |  |

^a^ Columns 1 to 2 represents the prognosis-related features and corresponding genes. And Columns 3 to 4 and 5 to 6 represents the results of Univariate Analysis and Multivariate Analysis, in which

hazard ratio (HR) and statastical significance (P value) were included.

**^$^** The features used in multivariate analysis were statistically significant features derived from univariate analysis. *p<0.05, **p<0.01, ***p<0.001

**Supplementary Table 11. Prognosis-related features with reported corresponding drugs^a^.**

| *feature* | *gene* | *drug* | *indication* |
| --- | --- | --- | --- |
| cg02654360 | PDE3B | DB01640(Experimental) | unknown |
|  |  | DB01970(Experimental) | unknown |
|  |  | DB07954(Experimental) | unknown |
| cg18421529 | MRAS | ~ | ~ |
| cg19585103 | SKAP1 | ~ | ~ |
| cg23835677 | FGFR3 | DB00039(Palifermin) | oral mucositis |
|  |  | DB05014 (XL999) | cancer/tumors (unspecified) lung/solid tumors |
|  |  | DB06589 (Pazopanib) | renal cell cancer/soft tissue sarcoma |
|  |  | DB09078 (Lenvatinib) | thyroid cancer |
|  |  | DB09079 (Nintedanib) | idiopathic pulmonary fibrosis |
|  |  | DB12010 (Fostamatinib) | chronic immune thrombocytopenia |
|  |  | DB12147 (Erdafitinib) | metastatic urothelial carcinoma |
|  |  | DB08901 ( Ponatinib) | chronic myeloid leukemia |
| chr7:127894457-127897682:+ | LEP | ~ | ~ |
| GAD2 | GAD2 | DB00142 (Glutamic Acid) | schizophrenia |
|  |  | DB00114 (Pyridoxal Phosphate) | nutritional supplementation |
| HTR2C | HTR2C | DB00574 (Fenfluramine) | exogenous obesity |
|  |  | DB00246 (Ziprasidone) | Schizophrenia and Bipolar I disorder |
|  |  | DB00334 (Olanzapine) | schizophrenia and bipolar I mania |
|  |  | DB00370 (Mirtazapine) | depressive disorder |
|  |  | DB00420 (Promazine) | moderate and severe psychomotor agitation |
|  |  | DB00777 (Propiomazine) | insomnia |
|  |  | DB00805 (Minaprine) | depression |
|  |  | DB01224 (Quetiapine) | Schizophrenia/related psychotic disorders |

^a^ Column 1 represents the features participate in the combined pathways illustrated in Figure 4 and Figure 5. Column 2 represents the corresponding genes of each features. Column 3 and 4 represents the drugs and corresponding indication.
